# Accounting for instrument resolution in the pair distribution functions obtained from total scattering data using Hermite functions

**DOI:** 10.1107/S1600576725004340

**Published:** 2025-07-22

**Authors:** Shaojie Wang, Min Gao, Yinze Qin, Sijie Zhang, Lei Tan, Martin T. Dove

**Affiliations:** a Institute of Atomic and Molecular Physics Sichuan University Chengdu Sichuan 610065 People’s Republic of China; b CrystalMaker Software Ltd Centre for Innovation and Enterprise Oxford University Begbroke Science Park Woodstock Road Begbroke Oxfordshire OX5 1PF United Kingdom; c China Spallation Neutron Source Institute of High Energy Physics Chinese Academy of Sciences 1 Zhongziyuan Road Dongguan 523803 People’s Republic of China; d College of Physics Sichuan University Chengdu Sichuan 610065 People’s Republic of China; e School of Mechanical Engineering Guizhou University of Engineering Science Xueyan Road Bijie Guizhou 55170 People’s Republic of China; f Department of Physics School of Physics and Mechanics Wuhan University of Technology Wuhan Hubei 430070 People’s Republic of China; g School of Physical and Chemical Sciences Queen Mary University of London Mile End Road LondonE1 4NS United Kingdom; DESY, Hamburg, Germany

**Keywords:** pair distribution functions, total scattering, resolution, Hermite functions

## Abstract

The use of Hermite functions to describe pair distribution functions (PDFs) from total scattering data was previously proposed by Krylov & Vvedenskii [*J. Non-Cryst. Solids* (1995), **192–193**, 683–687]. Hermite functions have a suitable form for describing both the total scattering data and the PDF, and have the useful feature that they are eigenfunctions of the Fourier transform operation. We demonstrate that, by fitting Hermite functions to total scattering data, it is possible to take into account the effects of experimental resolution when deriving the PDF from the scattering data. This is particularly advantageous for neutron time-of-flight data, where different banks of detectors have different resolution functions and the resolution widths vary with the size of the scattering vector. A number of technical points are discussed and illustrated using examples of synthetic data, including both amorphous and crystalline materials. These include a solution to the problem of handling the sharp Bragg peaks, and how to scale the scattering function and PDF to match the scale of the Hermite functions. A number of examples using real scattering data, both synchrotron X-ray and spallation neutron data, are also shown. To account for uncertainties in the levels of the scattering functions, we have modified a method of Billinge & Farrow [*J. Phys. Condens. Matter* (2013), **25**, 454202] to remove backgrounds by fitting with low-order orthogonal (Chebyshev) functions.

## Abstract 2

Hermite functions are used to represent pair distribution functions from total scattering data in order to allow the effects of resolution to be taken into account.

## 1. Introduction

A measurement of the total scattering of a material (whether fluid, glass or crystal) contains information about the short-range order in the material through the pair distribution function (PDF), which is obtained as the Fourier transform of the total scattering intensity. Specifically, this is given by 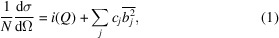
where the summation is over the atom type *j*, *N* is the number of atoms in the material, dσ/dΩ is the differential scattering cross section, 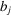 is the scattering length (scattering factor) of atoms of type *j* and 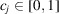 is the proportion of atom type *j*. The second term, which has constant value, is known as the ‘self-scattering term’.

The scattering function for coherent scattering, 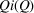, is related to the PDF 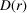 via the pair of Fourier transforms 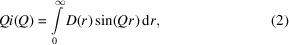

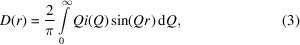
and the function 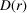 is related to the individual PDFs 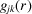 through 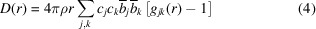
where ρ is the number density, that is, the number of atoms per unit volume [see, for example, Keen (2001 ▸), Peterson & Keen (2021 ▸) and Dove & Li (2022 ▸)].^1^ Because each individual PDF has the limiting value 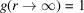, we have 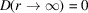. In the other limiting case we have 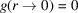, so it follows from equation (4) that 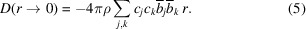
Similarly, the limiting values on the scattering functions are 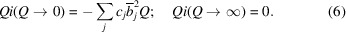
These limiting values are given by Keen (2001 ▸), Peterson & Keen (2021 ▸) and Dove & Li (2022 ▸), except that there is a common practice to scale the scattering function 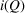 by the value of the self term, so that the limiting value of 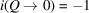, in which case the normalized scattering function will vary as 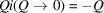. These limiting values are true regarding the coherent scattering due to interference between pairs of atoms within the extended sample. In the case where surfaces and interfaces are important, there will be small-angle scattering in the experiment that is not accounted for from the PDF as defined here. Furthermore, there is an additional term due to the compressibility (Keen, 2001 ▸), but this is of no practical importance in most cases. Typically, in the case of neutron scattering, the contribution to the overall scattering from incoherent scattering is removed in the data processing stage.

These limiting forms of 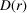 and 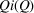, and the Fourier relationships between the two functions, are the key factors that motivate the choice of method described in the present paper.

The technical issue is how to obtain the best Fourier transform of a set of total scattering data, whether from neutron or X-ray radiation. For data obtained from a single set of detectors, and without paying any regard to the effects of resolution, this can be a relatively straightforward procedure, provided that all corrections from extraneous contributions to the measurement have been made. However, when data are obtained from different sets of detectors, as in modern instruments at spallation neutron facilities, and with each having a different resolution function, the procedure is not straightforward. Indeed, the common approach is to ignore the effects of instrument resolution and simply merge the data from different banks of detectors into one overall experimental 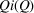 function. *Ad hoc* corrections can be made to handle misalignments of the background levels in measurements from different banks of detectors.

One approach that enables resolution to be taken into account is the inverse modelling of the Fourier transform, where a trial PDF is constantly modified until its Fourier transform convolved with the resolution function is in best agreement with the original data. The Monte Carlo version of this approach was first developed by Soper (1990 ▸) and Pusztai & McGreevy (1997 ▸). This approach was further developed by Tucker *et al.* (2001 ▸) for data obtained from the new generation of multi-detector instruments at spallation neutron sources, taking explicit account of the instrument resolution function. The problem with this approach, however, is that the transformation process takes a long time, whereas one frequently wants to see the Fourier transform virtually instantaneously.

In this paper we discuss an alternative approach for the analysis of the Fourier relations of equations (2) and (3) for total scattering data from single-detector or multi-detector total scattering instruments. This is based on the use of Hermite functions, as first introduced for this purpose by Krylov & Vvedenskii (1995 ▸). These authors pointed out that sums of Hermite functions are particularly appropriate for providing an analytical description of both of the functions 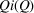 and 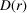, because the limiting behaviours of both functions, as described earlier, are met by the Hermite functions. Furthermore, two other particular advantages of Hermite functions are that they are orthogonal, so that fitting to data can be robust, and that they are the eigenfunctions of the Fourier transform operation. The latter point means that we can avoid performing explicit Fourier transforms in converting total scattering data to the PDF, meaning that the PDF is free from the constraints of the Fourier transform operation (*e.g.* that the range of values of *r* in the PDF is determined by the range of values of *Q* in the total scattering data).

The examples shown by Krylov & Vvedenskii (1995 ▸) were for data on liquid samples obtained from an earlier generation of total scattering instruments, with a restricted range of *Q* up to 12 Å^−1^. On the other hand, modern instruments at spallation neutron sources (for example) can routinely give data to values of *Q* up to 50 Å^−1^. Here we revisit the use of Hermite functions for the analysis of total scattering data, particularly as this approach can provide a fast alternative to the use of inverse methods to take account of instrument resolution when performing the Fourier transform of 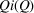 measured on different banks of detectors to obtain 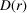.

## 2. Formalism

### 2.1. Hermite functions

The fundamental idea here is that it may be possible to represent a function 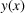 as a sum of Hermite functions (Celeghini *et al.*, 2021 ▸) (readers may recall meeting Hermite functions as the eigenfunctions of the Schrödinger equation for the harmonic oscillator), 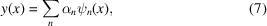
where 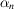 is a coefficient. The Hermite functions 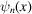 are defined as 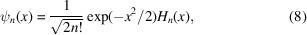
where the Hermite polynomials 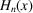 can be obtained from a generating recursion function (Wikipedia, 2022 ▸; Wolfram Mathworld, 2022 ▸): 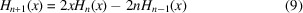
with the first terms given as 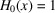 and 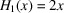. Thus the generating recursion function for the Hermite functions is 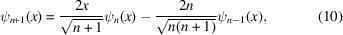
where 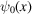 and 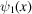 can be obtained from 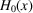 and 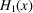 and equation (8). The odd-*n*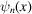 functions only contain terms with odd powers of *x*, and the even-*n* functions only contain terms with even powers of *x*. Hence we have the inversion property 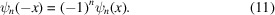


Krylov & Vvedenskii (1995 ▸) pointed out that the odd-*n* functions have a useful form for representing 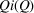 and 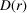. In the low-*x* limit, the odd-*n* terms have the form 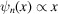. Beyond the low-*x* limit the 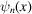 functions oscillate around zero. In fact, each odd-*n* function has 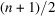 extreme points, with the last extreme point being the largest maximum. After the last maximum 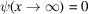.

### 2.2. Fourier and sine transforms

The important point here is that the Hermite function is an eigenvector of the Fourier transform operation (Wikipedia, 2022 ▸). Specifically, the Fourier transform of a Hermite function is related to the original function by 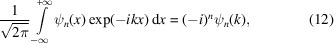
where *k* is the wavevector. The sine component of the transform, separately evaluated over the ranges 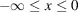 and 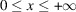, is 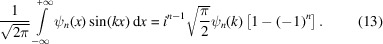
Clearly the terms with even powers of *n* are zero, so we only consider the odd-order terms. Thus we can write the sine transform as 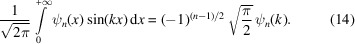
Hence we see that the sine transform has the same form as the initial function except that alternate terms in *n* have a change of sign (positive sign for *n* = 1, negative sign for *n* = 3, and so on). It is this easy way to set up the sine transform that makes Hermite functions so useful.

### 2.3. Application to the transformation of total scattering data

From the previous discussion, we can expand the scattering function 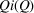 as an odd series of Hermite functions. Note that the range in *x* over which the Hermite functions have significant values is bounded. For applications where the range of data is arbitrary, this is an important limitation. The simplest approach is to use a scaling parameter, so that we can write 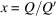 and hence 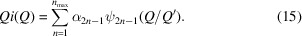
We discuss the issue of useful estimates for 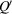 below.^2^

We can separate the series into two parts: 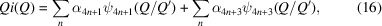
where the summations begin with the terms for 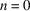. The sine transform according to equation (14) is then obtained by a simple change in sign of the second part: 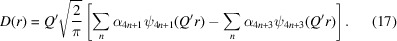


The value of equations (16) and (17) is that, if we can define the coefficients 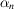 in one space, the Fourier transform is easily defined. For our key application, namely to be able to handle instrumental resolution, it is possible to convolve equation (16) with a resolution function – indeed, one can set up equation (16) for several banks of detectors, each with a different resolution function. One can then use the best set of values of the coefficients 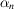 obtained by fitting to the experimental scattering function measured by different banks of detectors, and form the resultant 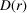 free of the effects of resolution via equation (17).

As an aside, we have recently demonstrated that the even-order Hermite functions can be used to perform the cosine Fourier transforms of time autocorrelation functions obtained from molecular dynamics simulations (Li *et al.*, 2025 ▸). In that application we made use of some of the points of implementation which will be discussed here next.

## 3. Application of the Hermite function formalism

### 3.1. Range of distances within the PDF

The range of values of 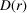 obtained from this analysis is set by the range of the experimental data for 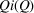 via 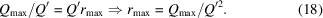
From the graphs presesnted by Krylov & Vvedenskii (1995 ▸), where 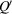 = 1 Å^−1^, it is clear that 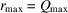 in terms of magnitudes. If the user defines the value of 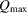 as given by their data, and the value of 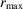 required for their application (and for applications such as in the reverse Monte Carlo method, the practical value of 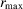 will be set by the chosen size of the atomic configuration), from equation (18) the useful value of 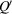 is defined as 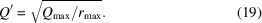
For the general case, where we expect to use values of 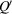 ≠ 1 Å^−1^ (usually 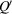 > 1 Å^−1^), we make use of the fact that the position of the last maximum in 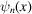 scales closely as 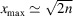 (the factor is actually 1.956 rather than 2). Thus, for the Hermite functions to fill the range of 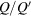, we will need to take them to a maximum value for *n* of 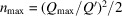. From equation (18) we have 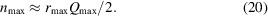
Equations (19) and (20) enable us to define the values of 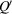 and 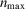 to use. For example, for 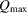 = 50 Å^−1^, we need to include Hermite functions up to 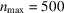 in order to obtain 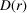 for 0 < *r* < 20 Å. In this case, we have 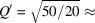 1.6 Å^−1^.

Krylov & Vvedenskii (1995 ▸) applied the method of Hermite functions to total scattering data from liquid Ge, Au and Cu, with 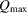 of 12, 7.8 and 7.9 Å^−1^, respectively (hence with corresponding values of 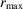, since effectively 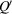 = 1 Å^−1^). The scattering functions were fitted using polynomials up to orders *n* = 73, 31 and 33, respectively; note that Krylov & Vvedenskii (1995 ▸) actually quote values of 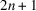. Their scattering functions did not contain a lot of sharp detail. On the other hand, with data obtained to much higher values of *Q* and sharper detail, it will be necessary to use many more functions.

### 3.2. Effects of cut-offs in the data

The form of the Hermite functions – example graphs are given in Wikipedia (2022 ▸) and Wolfram Mathworld (2022 ▸) – involves a reasonably sharp cut-off slightly above the position of the last maximum. This means that Hermite functions do not permit any extrapolation of the experimental data beyond the range of measurement, nor do they help avoid effects of data truncation with the Fourier transform. The truncation ripples produced by the Fourier transform are inherently built into equations (16) and (17).

In fitting the scattering data, we follow the common practice of multiplying the raw 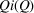 data by a single Lorch function (Lorch, 1969 ▸; Lorch, 1970 ▸) – otherwise known as the Lanczos filter (Lanczos, 1956 ▸; Duchon, 1979 ▸) – in order to reduce the truncation ripples in the resultant PDF: 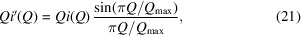
where 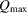 has been defined above. This has the effect of broadening the peaks in 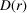 through convolution with a top hat function with width determined by the size of 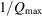. This filter also has the effect of reducing the contribution of noise in 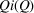 at higher values of *Q*; generally the noise on 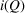 increases with *Q*, and is further magnified in 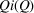 by the multiplication by *Q*.

### 3.3. Fitting procedure for crystalline materials

Crystalline materials have the added feature of Bragg peaks, which in kinematic scattering are Dirac delta functions convolved with the resolution function. If we are to model the 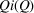
function prior to the effects of resolution, the Hermite functions will not be able to describe the Bragg peaks adequately.^3^

A simple solution to this problem is to note that we will only require – and indeed can only obtain – the 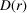 function to some maximum value of *r*. In effect, we are interested in the complete 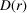 function multiplied by the box function, which is defined to be non-zero only over the range of values of 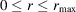. This is a common problem in comparing a 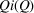 function obtained from a transformation of a model 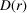 function defined over a finite range of values of *r* with experimental data. The solution in the standard reverse Monte Carlo method is to convolve the experimental 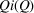 function with the Fourier transform of the box function prior to comparison with the Fourier transform of the bounded model 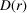 function. Thus, in order to handle Bragg peaks, our approach is to perform this convolution with the experimental data 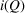 prior to fitting with the Hermite functions (Nield *et al.*, 1992 ▸; McGreevy, 2001 ▸):
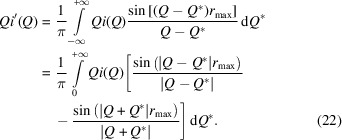
The effect of this convolution, as we will see below, is that the PDF is only defined for *r* up to the value of 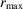 as selected by the user. In practice, this is rarely a problem.

The result of this is an overall scattering function from which resolution has been removed but to which artificial broadening accompanied by ripples has been added. This may appear to be a bad thing but, in truth, the most significant quantitative use of the scattering function is within the reverse Monte Carlo (RMC) method. And, in this case, this broadening with artificial ripples is added to the scattering data prior to fitting within the RMC method to account for the finite range of the PDF permitted by the size of the configuration. This point has been discussed by McGreevy (2001 ▸), with an example shown in his Fig. 4. Thus, if the value of 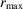 is selected so that it is consistent with the subsequent use in an RMC simulation, the scattering function generated by this method is exactly prepared for use in the RMC fitting.

## 4. Basic implementation with examples using synthetic data

### 4.1. Implementation

The main task is to obtain the values of 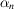 in equation (16) by fitting to the experimental 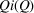 function; the function 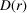 can be reconstructed via equation (17). For the development work we have written a script using MATLAB (MathWorks, 2022 ▸). For the efficient construction of Hermite functions we used a function developed by Polkinghorne (2022 ▸), who adopted a method of Clenshaw (1955 ▸). We used a number of MATLAB functions to make the basic implementation easier and faster to achieve. We developed a version of the routine to construct the Hermite functions in C and used the MATLAB interface to C programs (*mex*) to speed up this part of the calculation. We have also developed a separate Fortran version, but here we report results from the MATLAB implementation. The MATLAB software, here called *HermitePDF*, has been made available in a web repository (see Section 10).

The user must supply a file containing information needed to control the analysis (quantities such as the values of 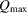 and 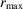) and one or more files containing the scattering data. The first file is in the format of keyword/value pairs, and some details regarding this file are given in Appendix *A*[App appa]. The values of the set of coefficients 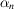 in equation (16) are obtained by fitting to the 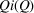 data, and then the transformation described in equation (17) can be simply constructed without the need to perform an explicit sine transform.

We tested the underlying ideas using synthetic data, firstly for an amorphous material and then for a few crystalline materials. In each case a synthetic PDF was converted to the scattering function 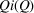 by the standard transformation, equation (2). To avoid truncation ripples in the synthetic 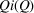 data, we applied a Lanczos filter (Lanczos, 1956 ▸; Duchon, 1979 ▸) to the PDF data: 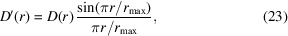
and then created the synthetic 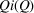 function via transformation of 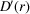.

### 4.2. Testing with synthetic data 1: fitting to the scattering function for amorphous silica without a resolution function

We generated a PDF for a configuration of amorphous silica containing 4096 formula units from a molecular dynamics (MD) simulation. This was done by first creating a random network of tetrahedral sites occupied by silicon atoms using the WWW method (Wooten *et al.*, 1985 ▸; Barkema & Mousseau, 2000 ▸); secondly, adjusting the linear dimensions of the configuration to create the correct density for amorphous silica; thirdly, adding oxygen atoms between neighbouring silicon atoms; and fourthly, relaxing the model using the *DL_POLY* MD code (Todorov *et al.*, 2006 ▸) with the potential energy function of Tsuneyuki *et al.* (1988 ▸). From the configuration we obtained the partial PDFs, which were used to form the PDF 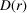 and then transformed to the scattering function 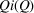 with values of *Q* up to 50 Å^−1^.

**Figure 1:**
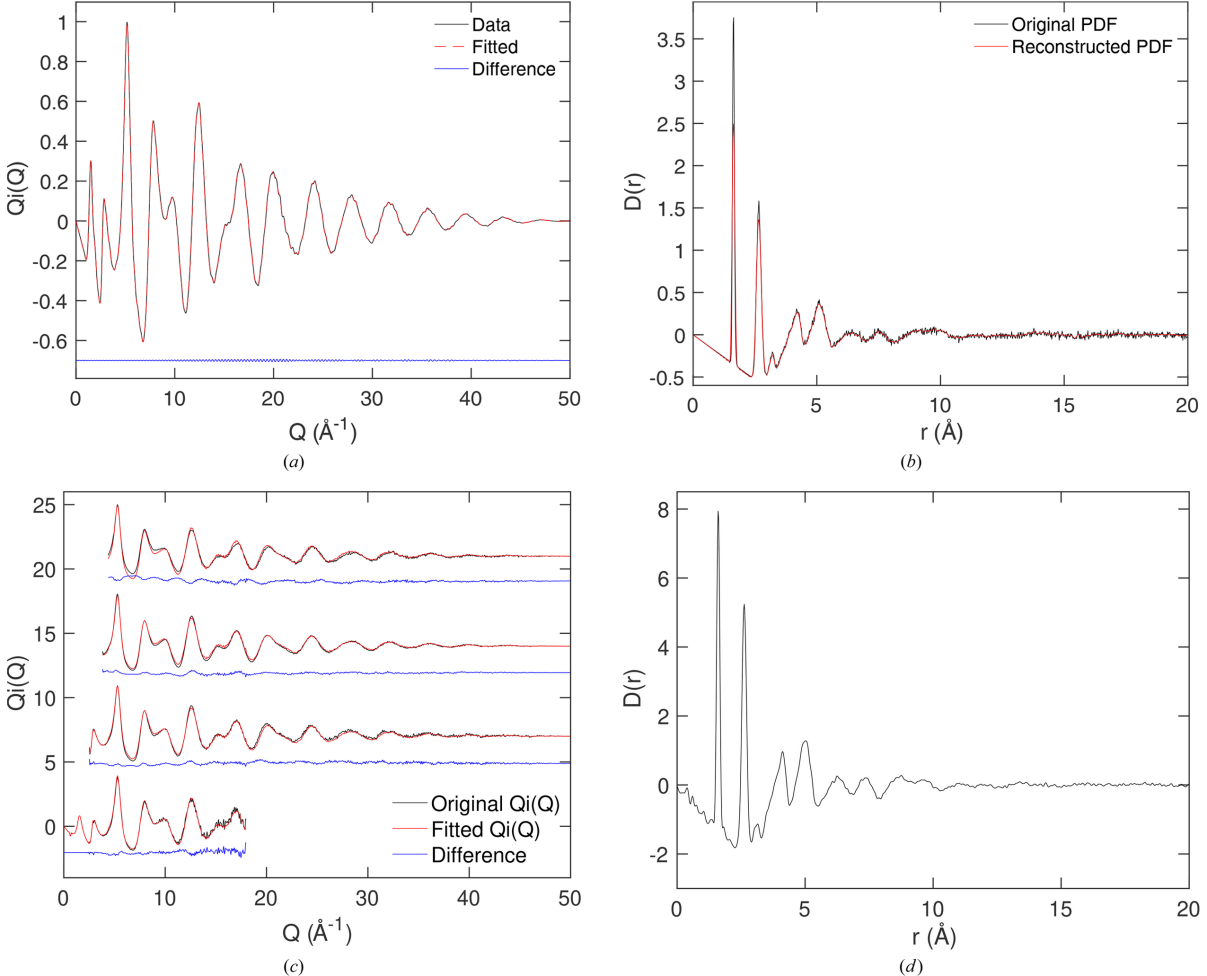
(*a*) The fitted 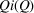 function for the model amorphous silica, with red showing the fitted curve and black (which is almost completely overlapped by the red curve) showing the simulated data. (*b*) The resultant PDF 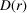 from transform of the fitted simulated 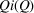 function shown as red, with black showing the original PDF from the simulation configuration. (*c*) The fitted 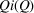 functions for experimental measurements of amorphous silica, showing fitting for four banks of detectors after removal of extraneous scattering not accounted for in the data correction/normalization procedure. (*d*) The resultant PDF 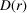 from transform of the fitted experimental 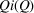 function.

The results are shown in Fig. 1 ▸; the fitted 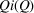 function is shown in Fig. 1 ▸(*a*), and the resultant 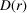 is shown in Fig. 1 ▸(*b*). The fit to the scattering function is extremely good, and the resultant PDF obtained by recombining the Hermite functions agrees with the original simulated PDF except in one point of detail. The resultant PDF is slightly broader than the original PDF, as seen in the first peak, because of the use of the Lanczos/Lorch modification function [equation (21)] to minimize the effects of an upper cut-off in the value of *Q*. In all other regards, the agreement between the original and reconstructed PDFs is extremely high.

Fig. 1 ▸(*c*) shows, for comparison, experimental data for neutron scattering from amorphous silica, obtained using four banks of detectors. The data normalization was good, but we used the methods described later in Section 7.1 to remove some residual extraneous scattering. The resultant PDF 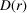 is shown in Fig. 1 ▸(*d*). This fitting will be discussed later (Section 7.3).

### 4.3. Testing with synthetic data 2: fitting to the scattering functions for crystalline phases without a resolution function

We computed PDFs for a range of crystalline materials from lattice dynamics calculations using empirical potentials, adoting the method described by Cope & Dove (2007 ▸) as implemented in the *GULP* lattice simulation code (Gale, 1997 ▸; Gale & Rohl, 2003 ▸). Our main example here is for andalusite (Al_2_SiO_5_; space group *Pnnm*). We have also performed some demonstration calculations for calcite (CaCO_3_; 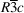), cristobalite (SiO_2_; 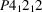), acetylene (*Cmce*) and quartz (SiO_2_; 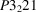), which are presented in the supporting information. In each case, the original PDF was generated to a distance of just over 50 Å, and the scattering function was created up to a value of the scattering vector *Q* = 50 Å^−1^ by direct application of equation (2). Truncation effects were handled in each case using equation (23).

**Figure 2:**
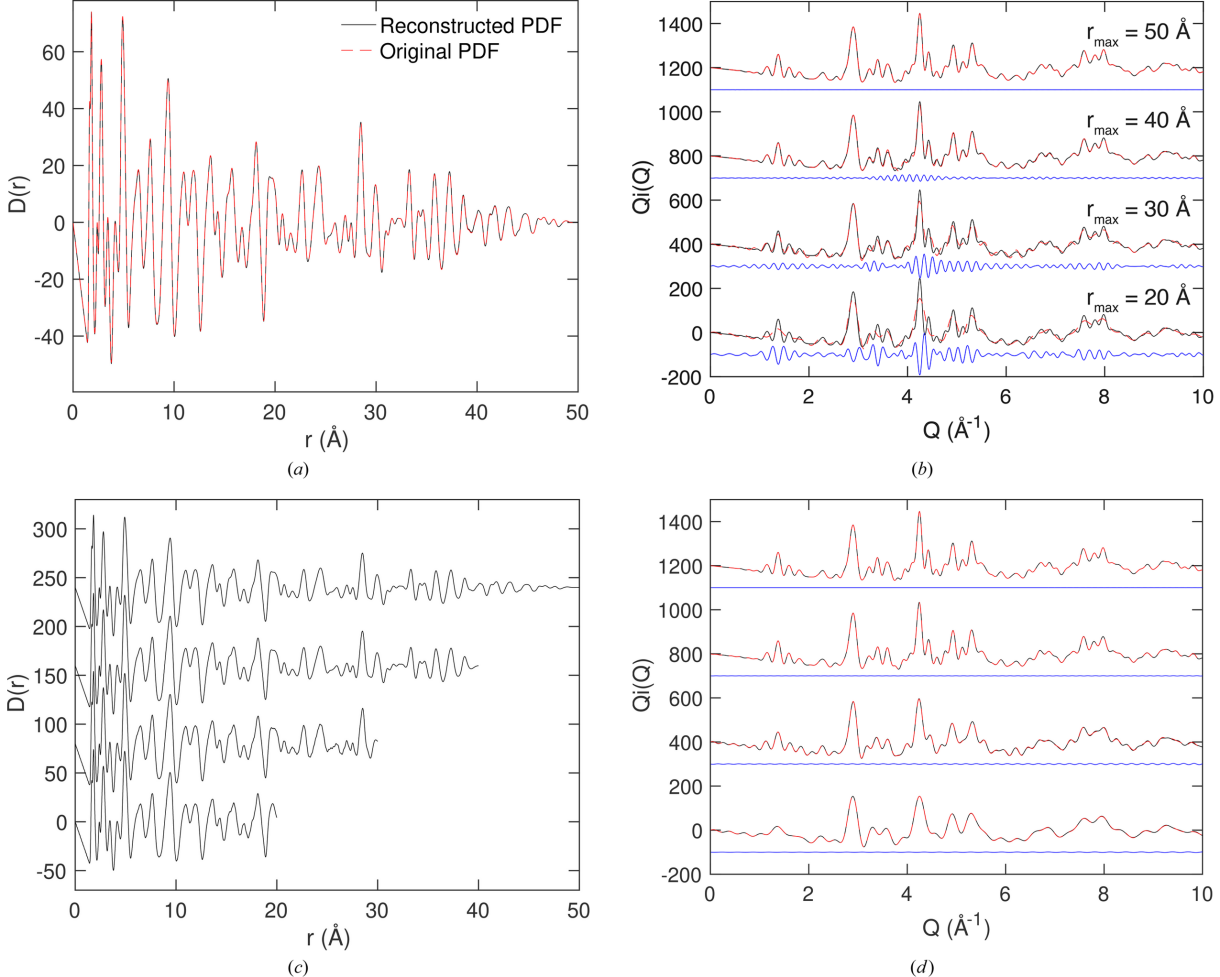
(*a*) Comparison of the PDF 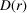 obtained by recombining the Hermite functions obtained by fitting to the simulated scattering function for andalusite, black curve, with the original PDF from which the simulated scattering function was obtained (dashed red curve). In this case the fitting was to the pure scattering function without convolution as defined by equation (22). (*b*) Fitted 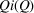 functions for different values of 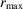, without using the convolution defined by equation (22). The black curve is the simulated function, red curves are the fitted functions and blue curves show the difference. (*c*) PDFs 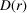 calculated from the scattering function convolved with the function of equation (22) for different values of 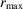 [defined by the range of the PDF, same order as in (*b*)]. (*d*) Fitted scattering functions using the convolution of equation (22) with the same values of 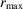 as in (*b*). The curves are as defined in pane (*b*).

The results for andalusite – generated using the transferable empirical model evaluated by Winkler *et al.* (1991 ▸) – are shown in Fig. 2 ▸. In Figs. 2 ▸(*a*) and 2 ▸(*b*), we show analysis for direct transformation of the simulated scattering function. In Fig. 2 ▸(*a*) we compare the PDF calculated from fitting the Hermite functions to the simulated scattering data with the original PDF from which the simulated scattering data were computed. The agreement for the case where we used 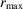 = 50 Å is extremely good across the full range of distances. In Fig. 2 ▸(*b*) we show the fitting to the scattering function 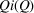 using different values of 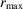 up to 50 Å. From equations (19) and (20) we can obtain the values of 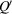 and the number of Hermite functions to use. The effect of smaller values of 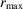 is to reduce the number of Hermite functions to be used. A smaller number might lead to a poorer fit, and this is what can be seen in Fig. 2 ▸(*b*).

Figs. 2 ▸(*c*) and 2 ▸(*d*) present results from where the scattering data are convolved with the effects of a finite range of *r* according to equation (22). Fig. 2 ▸(*c*) shows the PDFs calculated with different values of 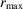. The actual PDFs are identical up to the limit of 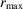; the limited number of Hermite functions appears not to have changed the details of the PDF. Fig. 2 ▸(*d*) shows the fitted scattering functions using the convolution of equation (22). The effects of the convolution can be seen as broadening of the features in the original scattering function, Fig. 2 ▸(*b*), and the formation of ripples at lower values of *Q*. It can be seen from Fig. 2 ▸(*d*) that for all values of 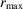 the fitting of the scattering function is extremely good, much better than without the convolution, as seen by comparison with Fig. 2 ▸(*b*).

**Figure 3:**
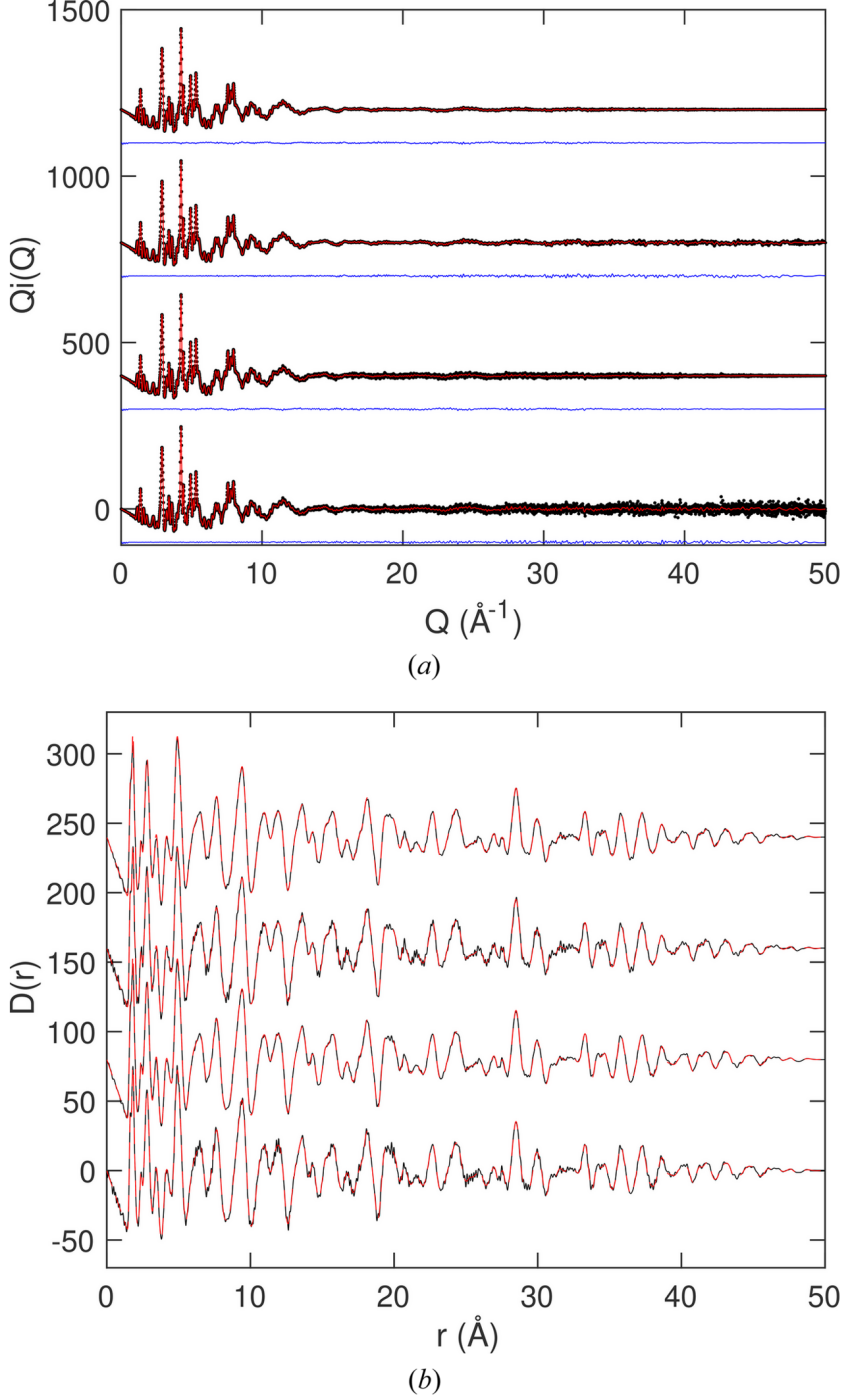
(*a*) The fitted 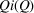 function for the simulated andalusite with inclusion of random noise, with red showing the fitted curve and black points showing the simulated data. From bottom to top we show data without either the Lorch correction [equation (21)] or convolution [equation (22)]; data with the Lorch correction but no convolution; data without the Lorch correction but with convolution; data with both the Lorch correction and convolution. The blue curves show the difference between the fitted function and the reference 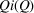 function with no added noise, given in Fig. 2 ▸(*b*). (*b*) The resultant PDF 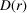 from transform of the fitted 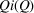 function shown as the black curve, with the red dashed curves showing the original model PDF.

Fig. 3 ▸ shows some calculations with added random noise, to the extent one might encounter in an experiment with low, but not recklessly low, counting times. It is assumed that the noise has constant amplitude in the 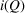 function across the range of *Q*, which means noise in 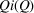 increases with *Q*. We examined four cases, namely with and without the Lorch correction [equation (21)], and with and without the correction for 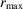 [equation (22)]. The primary effect of the Lorch correction is to broaden the peaks in the PDF, particularly noticeable for the peaks at low values of *r*. The effect of the convolution is actually to smooth out the noise in the scattering function. It can be seen from Fig. 3 ▸(*a*) that the fitted 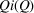 function smooths over the noise in the data. In consequence, there are few differences in the PDFs shown in Fig. 3 ▸(*b*), except that the noise gives a sharp truncation in the data at 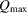 which is reflected as small ripples in the calculated PDF. Note that, because the pure scattering function is virtually zero in value at high *Q*, there were no truncation errors and there is no need to apply the Lorch correction.

The example calculations performed from synthetic data for andalusite shown here, together with the other examples given in the supporting information, show that the method of fitting Hermite functions to the scattering function and recombining them to yield the PDF works extremely well. In particular, the method seems to be robust in the presence of random noise, particularly so when using the convolution of the data to account for 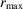 as given by equation (22), which has the effect of smoothing over noise without changing the Fourier transform.

Perhaps it is not surprising that this approach should work well given that it follows basic mathematics. The workflow involves fitting the coefficients 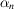 in equation (15); however, recombining the Hermite functions fitted in equation (16) to form the PDF 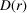 as in equation (17) involves changing the sign of half of the coefficients. If there are significant correlations between the values of the coefficients, there is a good chance that the process of recombining the Hermite functions as in equation (17) will break the Fourier transform. As seen in the results of this section, this has not proven to be the case, even when using a large number of functions (625 in the largest case). We are helped by the fact that the Hermite functions are orthogonal functions, which should act to minimize the degree of correlation between the parameters in the fitting.

## 5. Implementation with the effects of resolution

### 5.1. Method

The main point of this work is to be able to separate both the PDF and scattering functions from the effects of instrument resolution. The experimental scattering function for any set of data is represented by
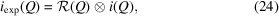
where 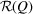 is the instrumental resolution function, which can be determined by fitting to the Bragg diffraction pattern in the scattering function. Typically, there are a set of standard functions for different types of instrument, whether neutron or X-ray, with parameters that can be fitted to the scattering data.

In order to account for resolution, the Hermite functions can be convolved with the experimentally determined 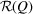 function, so that the experimental function described by equation (24) will be fitted by the function 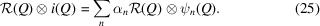
The deconvolved scattering function and PDF can then be reconstructed from the fitted values of the coefficients 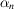 using equations (16) and (17), respectively.

Empirically, we have found that in many cases the functions describing the shapes of Bragg peaks in the total scattering data can be adequately described by a Gaussian function. This is true for most of the X-ray scattering data we have examined, even though sophisticated theoretical line shapes are available (Chernyshov *et al.*, 2021 ▸; Chernyshov *et al.*, 2024 ▸). Our example of SmB_6_ discussed below (Section 6) shows Bragg peaks across a wide range of scattering vectors, and these can be well fitted with a Gaussian function. Moreover, in this case, and other cases we have examined, the use of short X-ray wavelengths means that the range of scattering angles is small and thus the width of the Gaussian resolution function is practically independent of *Q*.

In the case of time-of-flight neutron scattering, we will have a different form of the resolution function 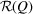 for different banks of detectors. Even though for Rietveld refinement from neutron time-of-flight data relatively sophisticated peak profile functions are available, in practice, again, we found that a Gaussian function can describe all but the data with highest resolution from the detectors with high scattering angles. In the case of time-of-flight neutron scattering, it is known that the resolution width 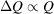.^4^ A *Q*-dependent width has easily been incorporated into our program.

The above discussion should not be taken to imply that our approach is constrained to work only with Gaussian functions of fixed width. We have programmed into our software, and tested, some other functions, notably the pseudo-Voigt function and a back-to-back pair of exponential functions convolved with a pseudo-Voigt function to model more accurately higher-resolution neutron time-of-flight data. In fact, any resolution function, including functions with *Q*-dependent parameters, can be used; the program has been designed to make it easy to implement different resolution functions, including those whose widths vary with *Q*.

We first test the method using synthetic scattering data (see the next section). Here we show the case where 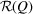 is a Gaussian function with constant width, and show the results for several widths, demonstrating that we always recover the same scattering function and PDF in each case. In the supporting information we show the results from the use of other resolution functions.

### 5.2. Testing with synthetic data 3: fitting to the scattering function of andalusite with a simulated instrument resolution

**Figure 4:**
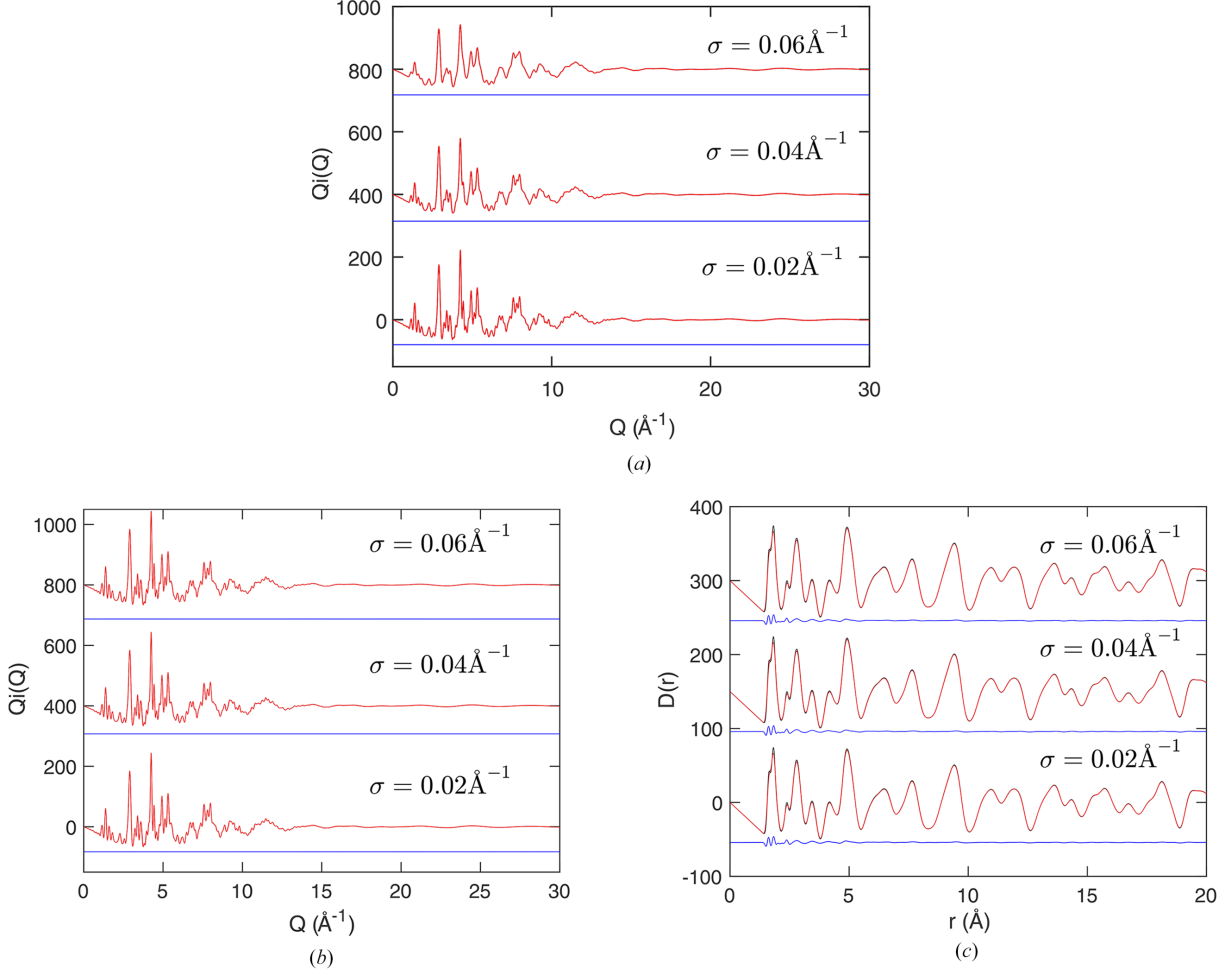
Demonstration of the reconstruction of the scattering function 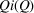 and PDF 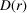 by accounting for resolution in the fitting of the scattering function with Hermite functions convolved with an appropriate resolution function, equations (24) and (25). (*a*) Three examples of broadened scattering functions 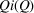 with fitting to the corresponding broadened Hermite functions. The black points representing the synthetic data are entirely overlapped by the red curves, which are the fitted functions. (*b*) The three reconstructed scattering functions 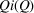 with the effects of the resolution function removed, showing close agreement in each case. The black points representing the synthetic data prior to the convolution with a resolution function (identical in each case) are overlapped by the red curves, which show the reconstructed scattering functions. (*c*) The reconstructed PDFs 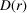 obtained with the effects of resolution removed (red curves) and compared with the initial data (black, identical in each case). Each plot gives the three values of the Gaussian width parameters, σ, equation (26). The blue curves in each case give the difference between the fitted or extracted functions and synthetic data.

Our test case is for andalusite. We took the scattering function discussed earlier [equations (24) and (25)], and convolved it with a Gaussian function defined as 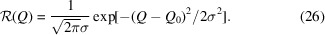
We compare the results for three values of the broadening parameter σ in Fig. 4 ▸. In each case we fitted the broadened scattering function as described in equation (24), Fig. 4 ▸(*a*), and then we used the fitted values of 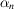 to reconstruct the resolution-free forms of 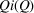 and 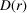. In Fig. 4 ▸(*b*) we compare the reconstructed 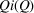 with the original synthetic function, and in Fig. 4 ▸(*c*) we compare the corresponding reconstructed resolution-free 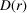 PDFs with the original PDF.

The key finding from Fig. 4 ▸ is that we have indeed recovered the original scattering function and PDF with the effects of resolution removed. The results for different values of σ are very consistent. The results presented in the supporting information show that the same is achieved with different forms of the resolution function.

**Figure 5:**
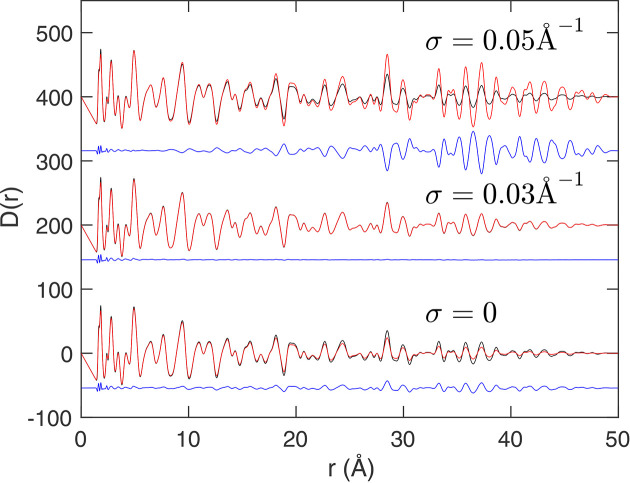
Demonstration of the effects of using the wrong parameters when fitting using a Gaussian resolution function [equation (26), values of the width parameter σ given in each plot], simulated using synthetic data for andalusite. The black curves represent the original synthetic 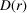 function for andalusite, the red curves show the reconstructed PDFs and the blue curves give the differences. The middle figure, σ = 0.03 Å^−1^, is for the correct value of σ.

We can ask an interesting practical question: what might happen if we use the wrong resolution function? This includes the case of using an effectively reasonable resolution function but with errors on the parameterization. We can imagine from the convolution theorem that, if the resolution function that we use is too narrow, we will see attenuation at higher values of *r*, as if we did not take account of resolution at all. We can also imagine that, if the resolution function that we use is too broad, we will see over-compensation for attenuation at higher values of *r*. This is demonstrated in Fig. 5 ▸. The data were generated using a value of σ = 0.03 Å^−1^ in equation (26). In the extreme case of σ = 0, that is, no account taken of resolution in the fitting, we see the attenuation in the reconstructed PDF. In the case of σ = 0.05 Å^−1^ the over-compensation is seen as a growth in the amplitude of the oscillations in the PDF at higher values of *r*. If the over-compensation is high, then the oscillations in the PDF will grow almost uncontrollably with increasing values of *r*. Effectively, the mathematical process is to divide the PDF by the Fourier transform of equation (26), which is of course another Gaussian. Since this will fall close to zero at higher values of *r*, if the value of σ used in the fitting is too large, the Fourier transform of 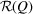 at higher values of *r* will be both small and smaller than it should have been, so the over-compensation will actually grow on increasing *r*. In practice, this over-compensation will serve as a practical warning when being used with real data.

## 6. Working with real data 1: X-ray scattering data from samarium hexaboride, SmB_6_, with good corrections and normalization

In this section we demonstrate how the methods outlined above can be applied to real (experimental) data. In this first part we consider data for which the corrections and normalization have been carried out successfully, with the new feature being the attempt to take account of the experimental resolution function.

X-ray total scattering data for SmB_6_ were obtained for temperatures between 20 and 300 K by Li *et al.* (2024 ▸) using the XPDF (I15-b) instrument at the Diamond synchrotron facility. Experimental details have been reported by Li *et al.* (2024 ▸). Data were corrected and normalized using a combination of the *Dawn* (Filik *et al.*, 2017 ▸) and *Gudrun-X* (Soper & Barney, 2011 ▸; Soper, 2011 ▸) software.

The scattering function of SmB_6_ has a significant contribution from the Bragg peaks, which are quite sharp and extend to high values of *Q*. It was found that the resolution function closely approximates a Gaussian function. The high *Q* resolution gives Bragg peaks with a full width at half-height of 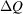
 = 0.052 Å^−1^ and a variation of less than 1% across the range 0.5 < *Q* < 30 Å^−1^. The constancy of 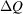 across the range of *Q*, at least where we can fit individual Bragg peaks, arises because with a small wavelength of the beam the range of scattering angles is small (λ = 0.161669 Å, scattering angle 0.75–45.4°). For these data, inclusion of the resolution function will give an attenuation of 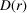 equal to 50% when *r* = 54 Å. At *r* = 15 Å the attenuation will only be 5%, and it will be 14% at *r* = 25 Å.

**Figure 6:**
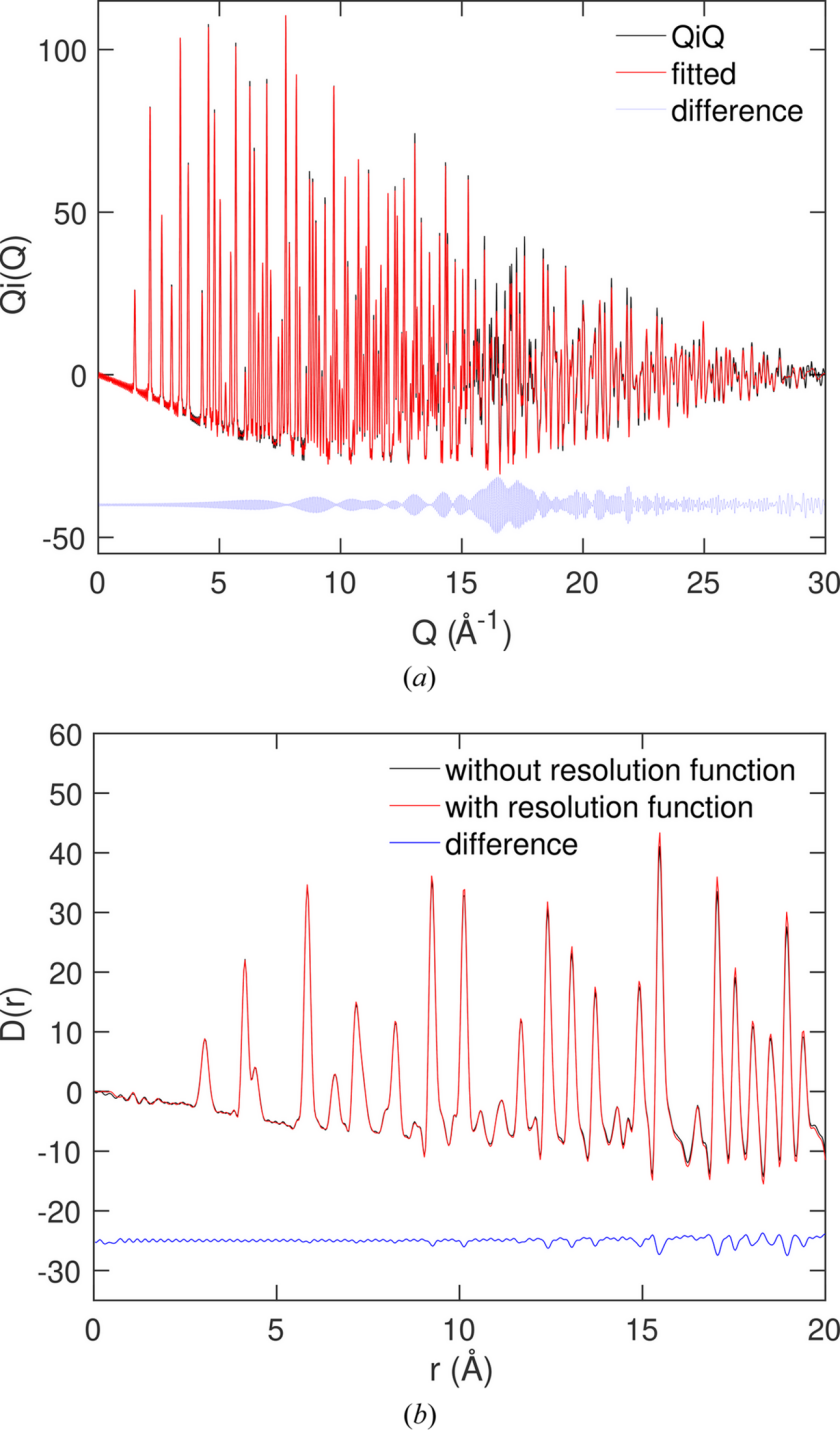
(*a*) The fitted 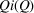 function for SmB_6_X-ray total scattering data, with red showing the fitted curve and black (which is almost completely overlapped by the red curve) showing the simulated data. (*b*) The resultant PDF 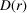 for SmB_6_ from transform of the fitted 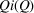 function shown as red, with black showing the original PDF from the simulation configuration.

We show the fitted scattering data in Fig. 6 ▸(*a*), together with the resultant PDF in Fig. 6 ▸(*b*). This is actually quite a strenuous test case because the Bragg peaks are sharp and there are many of them extending to very high *Q*. The small oscillations in the scattering function arise from the application of equation (22) to enable fitting of the Bragg peaks. In this case, as indicated above, the effect of accounting for resolution is small. However, the small attenuation of 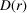 caused by neglecting resolution, increasing as *r* increases, can be seen in Fig. 6 ▸(*b*).

The result is a 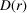 function that has no significant noise in its low-*r* part, with a linear decrease in the baseline, as expected. In this case, the peaks in the PDF are so sharp that the trace of the downwards-sloping baseline can be seen to extend all the way down to *r* = 20 Å.

## 7. Working with real data 2: data with unknown data corrections and normalization

### 7.1. Handling unknown contributions to the total scattering data

#### 7.1.1. Correcting data for unknown factors

A recurring problem in analysing total scattering is that researchers often cannot know that their total scattering data are free of extraneous contributions. Of course, many factors are known and therefore can be accounted for. Measurements of the empty sample container can be subtracted from the scattering data as a routine matter, provided that there is no noticeable attenuation of scattering by the sample or sample container. For X-ray scattering, data reduction/correction software such as *Gudrun-X* (Soper & Barney, 2011 ▸; Soper, 2011 ▸) and *Dawn* (Filik *et al.*, 2017 ▸) attempt to incorporate contributions such as Compton scattering and fluorescence, and to take account of attenuation of the beam by the sample container and the sample itself. As the example of SmB_6_ above shows, the data reduction/correction from synchrotron X-ray data can be very successful. However, there are cases where the corrections are clearly inadequate. Worst are cases where none of the corrections apart from the subtraction of the scattering from the sample container can be carried out, and where even that may not be completely accurate if no account can be taken of the effects of beam attenuation by the sample.

With regard to the problem of X-ray scattering, Billinge & Farrow (2013 ▸) pointed out that, for total scattering measured within a single total scattering spectrum, the extraneous scattering is likely to be a slowly varying function of *Q*, and as such the most significant effect will be seen as ‘noise’ in the low-*r* region of 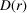. They suggested fitting a low-order polynomial to the total scattering data, which is designed to be insensitive to fast-varying features in 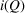 but which is also able to model the slow-varying features that give the discrepancy between the mean value of 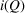 or 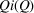 and zero. The rationale for this is that the total scattering data 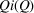 are known to form a function that oscillates around zero over most of the range of values of *Q*. A slowly varying function will be insensitive to rapid changes in 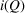 or 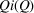 and will only be sensitive to an underlying ‘background’ that does not conform to the average of zero.

We initially explored this approach using neutron total scattering data with multiple banks, where the data processing was performed without the use of the Placzek correction (discussed below). The data from the different banks were widely different at higher *Q*, and in a way that became worse at higher temperatures. We found that a simple regular polynomial was not robust. However, by using orthogonal polynomials, such as the Chebyshev polynomials, we were able to bring the 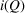 and 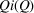 functions for all banks into consistency, both in the low-*Q* and high-*Q* limits and for all temperatures. Therefore in this work we have modified the approach of Billinge & Farrow (2013 ▸) by using Chebyshev polynomials of the first kind, 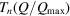, rather than regular polynomials. We will describe this in more detail below.

As Billinge & Farrow (2013 ▸) noted, this process effectively removes the absolute normalization from the data. Billinge & Farrow (2013 ▸) thought that normalization can be recovered by fitting the PDF to a model that includes an adjustable scale parameter, and therefore absolute normalization may not be necessary. This is true in such an approach, but there are cases where the absolute normalization is required. For example, if the first two to three peaks in the PDF are to be integrated to give coordination numbers, for example in studies of liquids or amorphous solids, absolute normalization is essential. This can be estimated from the initial slopes of both 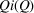 and 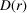 provided that the scattering data have been measured to sufficiently low values of *Q*, and provided most of the noise has been removed from the PDF at low values of *r*. To some extent, this will require critical attention by the user. We note that it is becoming increasingly common to simply disregard or discard the low-*r* part of 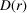, as it is considered to be inherently unreliable. We do not believe this approach to be necessary (Dove & Li, 2022 ▸).

In using Chebyshev polynomials to account for the background, we make use of the fact that 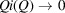 as 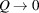. This means that we only use the odd-ordered Chebyshev polynomials, spanning the range 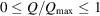.

We are not able to fit the Chebyshev polynomials at the same time as fitting the Hermite functions, because the two types of polynomial are not orthogonal to each other. Our strategy is to fit the Chebyshev polynomials as a first step, subtract the fitted Chebyshev polynomials and then use the modified scattering functions as the basis for fitting with the Hermite functions.

#### 7.1.2. Correcting neutron scattering data for unknown effects of inelasticity

When neutron data are collected at a spallation source with multiple banks of detectors, a total scattering spectrum is obtained for each bank, each having its own resolution function and a different range of values of *Q*. Typically, the banks with higher scattering angles will give scattering data that extend to the highest values of *Q*, but the data will not extend to the lower values of *Q*. The bank of highest scattering angle will also have the finest resolution in *Q*. When we compare data from different banks, whilst the effect of resolution can be seen, it is also often found that the ‘background’ levels of the corrected 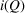 are not consistent. Ideally 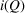 should oscillate around zero for higher values of *Q*, but often it is found that 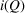 has a *Q*-dependent background (of positive or negative value), which in some cases can become more significant at higher temperatures, as mentioned above.

The measured scattering cross section, equation (1), contains both 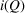 and a quantity known as the ‘self term’ [the second term in equation (1)]. Once all corrections for additional sources of scattering (from sample container, sample environment equipment and the basic instrument), beam attenuation (sample, sample container, sample environment equipment), incoherent scattering (in the case of neutron scattering), detector normalization, Compton scattering and fluorescence (in the case of X-ray scattering) have taken place (Howe *et al.*, 1989 ▸; Hannon *et al.*, 1990 ▸; Fischer *et al.*, 2006 ▸; Soper, 2011 ▸; Dove & Li, 2022 ▸), the self term is then subtracted. Ideally, this should yield a function 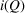 that has the right quantitative behaviour in the limits 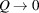 and 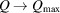, equation (6). In the case of neutron scattering, there is an important correction for the effects of inelastic scattering processes – the ‘Placzek correction’ – which is more significant for higher scattering angles and lighter elements (Soper, 2009 ▸). The Placzek correction is found to most affect the self term. If the Placzek correction is not applied, or applied ineffectively, this will account for the way in which background to the scattering function can vary with temperature. Note that a rigorous Placzek correction would require knowledge that is not available to the researcher (regarding the vibrational density of states). Often with data where the Placzek correction has been included in the data processing, we find a mismatch of a few per cent, or worse, in the values of the scattering functions at high *Q*. Dove & Li (2022 ▸) show a best-case example (their Fig. 9), and we investigate these data in this paper (Section 7.4).

The effects of an inadequate Placzek correction, or of none, being applied to neutron total scattering data can also be taken into account using the method of Billinge & Farrow (2013 ▸). However, unlike the case envisaged by Billinge & Farrow (2013 ▸), we typically have data for several banks of detectors, as noted above, with none covering the full range of values of *Q*. Our approach was to span the range of *Q* for each bank with the Chebyshev polynomials, ranging from zero to the maximum *Q* for each bank, and scaling the maximum order for each by the size of the extent of *Q*. That is, we used more higher-order Chebyshev polynomials for the higher-angle banks.

### 7.2. X-ray scattering from data without data correction and normalization: hematite, Fe_2_O_3_

**Figure 7:**
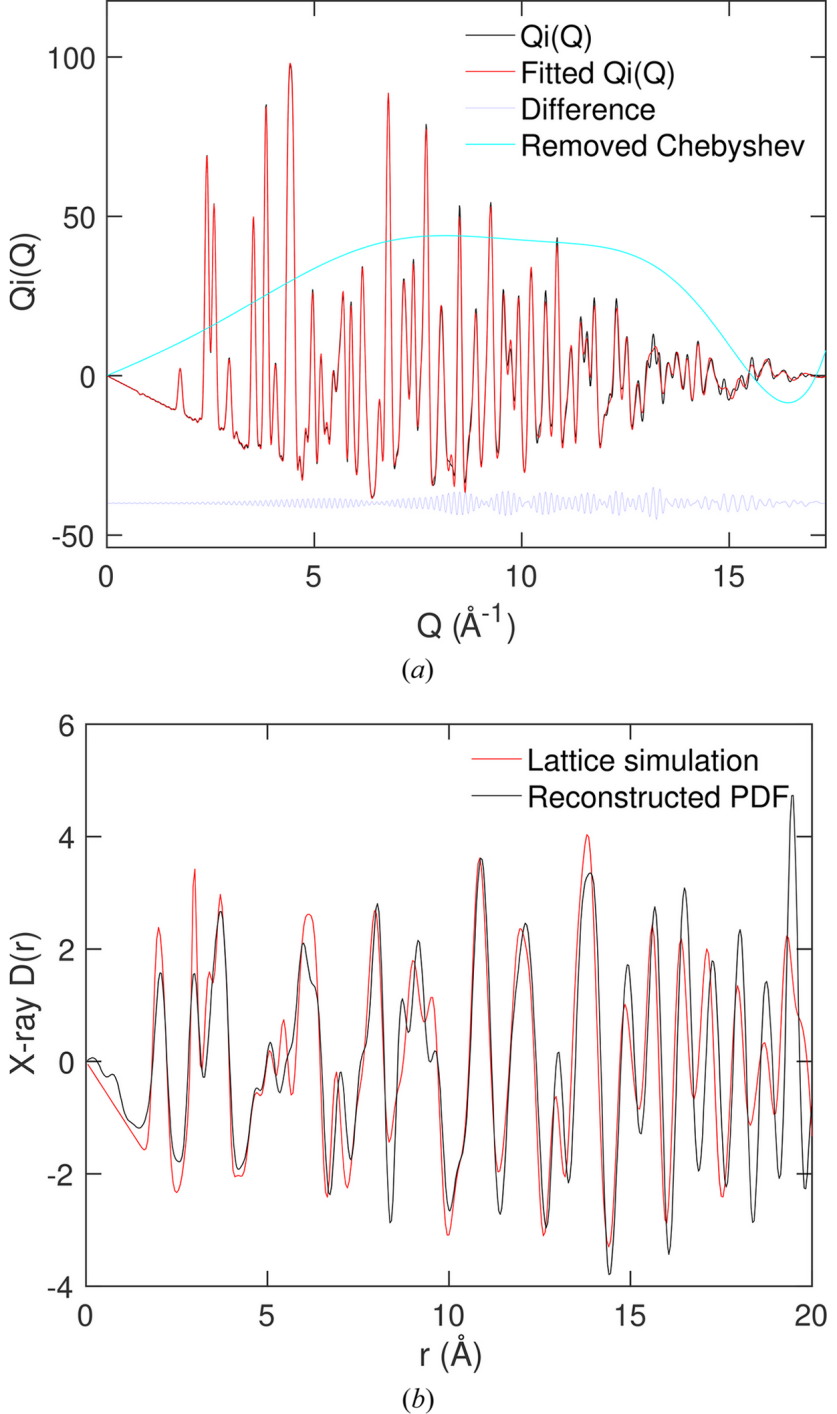
(*a*) The fitted 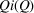 function for X-ray total scattering data from crystalline Fe_2_O_3_, with the red curve showing the fitted curve, the black curve showing the experimental data after removal of the effective extraneous scattering identified by fitting with low-order Chebyshev functions and the cyan curve showing the extraneous scattering that had been removed. (*b*) The black curve is the resultant PDF 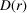 for Fe_2_O_3_ from transform of the fitted 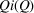 function and taking account of a Gaussian resolution function. The red curve is the result from the lattice simulation described in the text.

We have been presented with a number of X-ray total scattering data sets where there are clearly extraneous contributions to the total scattering that were not taken account of by the normal data reduction/correction processes. Here we show analysis of some of our own synchrotron X-ray total scattering data for crystalline Fe_2_O_3_ obtained at the Shanghai Synchrotron Facility (Fig. 7 ▸). Measurements were performed on the BL13SSW beamline, with X-ray beam energy of 50 keV and wavelength 0.2480 Å. The sample was contained within a thin-wall silica glass capillary tube. Measurement times for both sample and empty container were 10 min.

The initial data were obtained by extracting the one-dimensional scattering functions for both the container and sample + container together from the two-dimensional data taken from the flat-plate detector. We subtracted the scattering from the container from the sample + container data, and then converted the data from scattering angle to *Q*. We estimated that the useful range of values of *Q* is from 1 to 17 Å^−1^. The effects of the atomic electron density were approximately deconvolved from the scattering function by dividing the data by the average squared atomic scattering factor at each value of *Q*, as is normal practice with X-ray scattering data.

In Fig. 7 ▸(*a*) we compare the scattering function 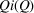 obtained after removal of extraneous scattering by fitting with Chebyshev polynomials with the fitted function, and also show the removed scattering. We show the resultant PDF 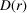 in Fig. 7 ▸(*b*), and compare with a calculation (discussed below). The PDF appears to be satisfactory, and the noise at low values of *r* is small enough that the downward slope in 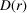 can be clearly seen. We did not optimize the number of Chebyshev polynomials to reduce the noise to a desired level.

As an aside, we note that in general application of the method of Billinge & Farrow (2013 ▸), employing regular non-orthogonal polynomials, the user is able to select the maximum order of the polynomial to best reduce the level of noise in the PDF at low values of *r*. We have noticed that sometimes users have not appreciated that the 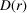 function has a downwards slope at low *r*, and users have optimized the correction so as to give a flat function with small oscillations around 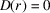, then allowing for a dip down to the first peak in 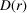. We suspect, but have not properly investigated, that the use of orthogonal polynomial functions rather than regular non-orthogonal polynomials may actually help preserve the general shape of 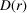 at low values of *r*.

The calculated PDF shown in Fig. 7 ▸(*b*) was formed using the PDF module (Cope & Dove, 2007 ▸) within the *GULP* lattice simulation program (Gale, 1997 ▸; Gale & Rohl, 2003 ▸) as used to form the synthetic data in Sections 4 and 5. Calculations were performed using the interatomic potentials of Bush *et al.* (1994 ▸) using a rigid-ion model. The overall PDF was formed by summing the partial pair 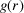 functions [equation (4)] and setting the scattering factors equal to the atomic number. A slight rescaling of the distance scale in the simulated PDF was performed to get a match of the distance scale – this rescaling is necessary partly to offset slight failings in the model and partly to account for errors in the calibration of the scattering data. We also rescaled the calculated PDF to approximately match the downward background slopes of the two PDFs, because no account was taken of absolute normalization in the conversion from raw data to scattering function. The experimental and simulated PDFs agree to a high degree in terms of the positions and relative intensities of the peaked features. The differences are consistent with likely inadequacies in the simple interatomic potential used in the simulation and the fact that the deconvolution of the atomic electron densities is approximate rather than exact. Furthermore, the effects of broadening of the peaks in the experimental data due to a relatively low maximum value of *Q* were modelled in the simulation by using a high temperature to increase the thermal broadening of the peaks in the PDF, which of course is itself not the same thing but is probably of lower consequence.

### 7.3. Neutron scattering data from silica glass with small residual normalization problems

We introduced the idea of fitting scattering data with Hermite functions by using synthetic data for amorphous silica obtained from computer simulation [Figs. 1 ▸(*c*) and 1 ▸(*d*)]. We now discuss how we have also fitted neutron scattering data from amorphous silica obtained from the ISIS Neutron and Muon Source (Tucker *et al.*, 2005 ▸). In this case we did not include the effects of resolution, because the features in the data are all much broader than the resolution function, but we have needed to account for a small residual inconsistency between the data from different banks using pre-fitting by Chebyshev polynomials. The results are shown in Figs. 1 ▸(*c*) and 1 ▸(*d*). There is a good, although not required, consistency between the simulated and experiment PDFs.

In our review paper on PDFs from neutron data (Dove & Li, 2022 ▸), we presented an analysis of these data using Hermite functions without taking account of the residual backgrounds to the data. In that instance we actually obtained a small noise peak in the PDF at very low values of *r*. We can see from Fig. 1 ▸(*d*) that our approach of pre-fitting with Chebyshev polynomials to bring the data from different banks into close alignment has automatically reduced the noise in the PDF at low *r*. Again, we have not attempted to optimize the number of Chebyshev polynomials.

### 7.4. Neutron scattering data from scandium fluoride, ScF_3_, with residual normalization problems

Our demonstration case is for neutron total scattering data obtained from the cubic material ScF_3_ obtained at the ISIS spallation neutron facility on the Polaris diffractometer (Smith *et al.*, 2019 ▸); the measurement and the data are described in detail by Dove *et al.* (2020 ▸). In this case, the data for the scattering function after full processing and correction using *Gudrun* (Soper, 2011 ▸) show a slight (7%) difference in the background levels at high *Q*, as shown in Fig. 9 of the review paper by Dove & Li (2022 ▸). This is due to slight inadequacies in the application of the Placzek correction, affecting the subtraction of the self term. These discrepancies are actually handled within *Gudrun* when merging data from all banks to perform the Fourier transform of the overall data to generate the PDF. This example is a relatively good case; we have examples where the mismatch is much larger.

**Figure 8:**
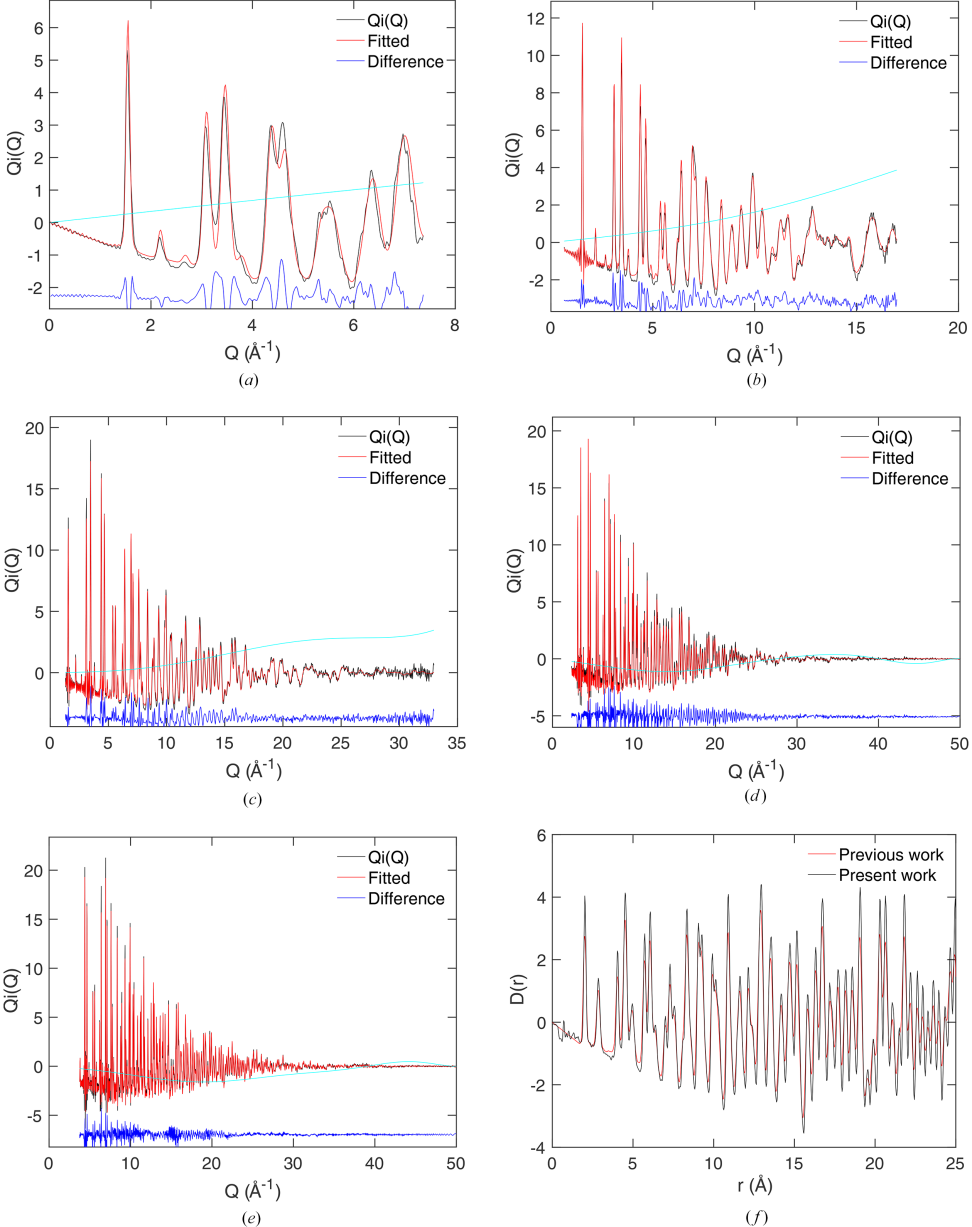
(*a*–*e*) Fitted neutron scattering function, 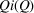, for ScF_3_ measured at a temperature of 10 K, showing data from five detector banks (Dove *et al.*, 2020 ▸). The black and red curves are the experimental data and fitted function, respectively, with the blue curves, displaced downwards, showing the differences. The cyan curves show the extraneous scattering removed by fitting Chebyshev polynomials. (*f*) The resultant PDF 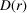 obtained by recombining the Hermite functions, black curve, compared with the PDF obtained from the same data reported earlier (Dove *et al.*, 2020 ▸; Dove & Li, 2022 ▸), red curve.

In this example we used the Gaussian resolution correction with peak width proportional to *Q*. Parameters were obtained by fitting Gaussians to several Bragg peaks in each data set, which was easy to do because the crystal structure is cubic, and therefore the Bragg peaks do not overlap significantly. The fitted 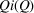 data are shown for each bank of detectors in Figs. 8 ▸(*a*)–8(*e*). In these figures we show the small corrections from the fitting by Chebyshev polynomials required to bring the data to a common level. The resultant PDF 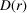 is shown in Fig. 8 ▸(*f*). It can be compared with the PDF obtained using the *Gudrun* software (Soper & Barney, 2011 ▸) as presented by Dove *et al.* (2020 ▸) and as used as an example by Dove & Li (2022 ▸).

The PDF shown in Fig. 8 ▸(*f*) is in remarkably good agreement with the data obtained from previous analysis (Dove *et al.*, 2020 ▸; Dove & Li, 2022 ▸). However, the PDF has now been corrected for the effects of resolution, which were ignored in the previous work and which is our main achievement here. The PDF obtained here has some residual noise at low *r*, but this is not significant and does not obscure the downwards slope of 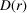.

### 7.5. Neutron scattering data with unknown data corrections: copper pyrophosphate, Cu_2_P_2_O_7_

Our final example concerns new data, for copper pyrophosphate, Cu_2_P_2_O_7_ (monoclinic symmetry), measured at the new MPI instrument at the China Spallation Neutron Source (Xu *et al.*, 2019 ▸; Xu *et al.*, 2021 ▸). Data were corrected for the major contributions of scattering and attenuation using in-house software, but no correction was made for inelasticity. As a result, the data from different banks show a significant mismatch in their levels (although of course the Bragg peaks have consistent positions), with a *Q*-dependent background in the scattering functions that clearly varied with temperature.

**Figure 9:**
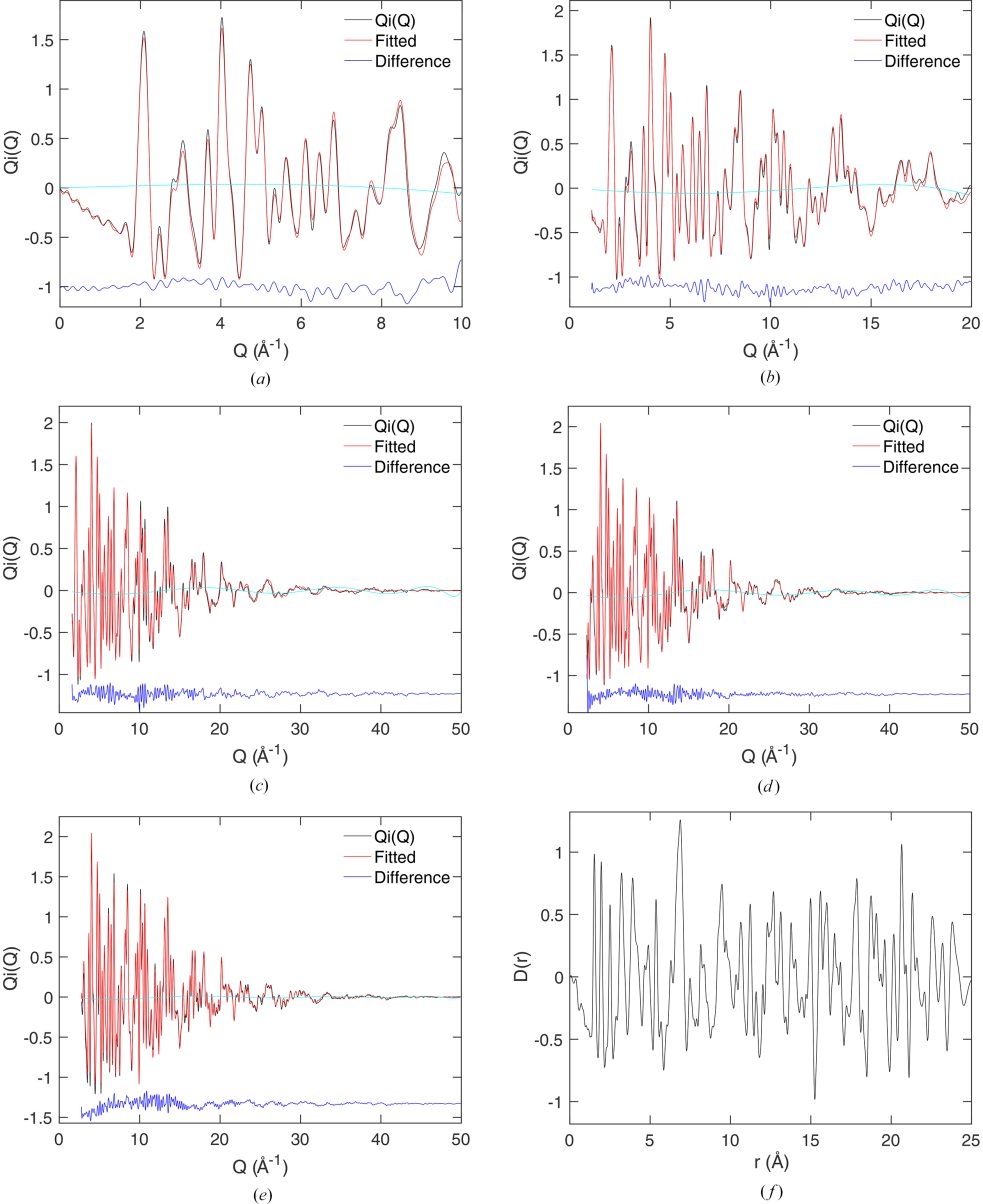
(*a*–*e*) Fitted neutron scattering function, 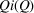, for Cu_2_P_2_O_7_ measured at a temperature of 20 K from five detector banks. The black and red curves are the experimental data and fitted functions, respectively, with the blue curves, displaced downwards, showing the differences. The cyan curves show the extraneous scattering removed by fitting Chebyshev polynomials. (*f*) The resultant PDF 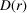 obtained by recombining the Hermite functions.

As for ScF_3_, we used a resolution function based on Gaussian functions with width in each detector that varies linearly with *Q*. Because the crystal structure of Cu_2_P_2_O_7_ is monoclinic, the Gaussian resolution functions were calibrated with a prior measurement of elemental crystalline silicon. The fitted total scattering data for different banks of detectors are shown in Figs. 9 ▸(*a*)–9(*e*), with instrument resolution taken into account. The resultant PDF 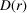 is shown in Fig. 9 ▸(*f*).

In this case, because the data have not been corrected for inelasticity, there was a significant mismatch of the levels of the scattering function across the range of values of *Q*, and particularly at higher values of *Q*. By fitting with Chebyshev polynomials, we have been able to remove this discrepancy and form a PDF taking account of the effects of resolution. There is no prior measurement with which to compare. This PDF also has a small amount of noise at low values of *r*, but again not significant enough to mask the downward slope of 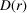.

## 8. Summary

In this paper we have reported the use of Hermite functions to describe total scattering data, as initially proposed by Krylov & Vvedenskii (1995 ▸). The original idea of Krylov & Vvedenskii (1995 ▸) was applied to data for liquids obtained over a relatively narrow range of *Q* values, giving a PDF over a similarly narrow range of *r* values. For many liquids this is not unreasonable, given that usually there is little structural order beyond the second shell of neighbours, and correspondingly little information in the scattering function at the higher values of *Q* that can now be achieved experimentally.

Our main objective is to use this approach to account for instrument resolution, particularly for the analysis of data obtained from the latest generation of instruments on time-of-flight spallation neutron sources, where different banks of detectors have markedly different resolution functions and where the width of the resolution function varies with *Q*. Our approach allows the application of the method for the wider range of *Q* that is now available, and we have also adapted the method to enable its application to crystalline materials showing sharp Bragg peaks. The method has allowed us to account for instrument resolution where the widths are either constant or varying with *Q*.

We have tackled the problem of uncertainties in the background levels of the scattering following the approach of Billinge & Farrow (2013 ▸). However, whereas Billinge & Farrow (2013 ▸) proposed fitting the slow variation in background using regular polynomials, we have used the orthogonal Chebyshev polynomials, which we have found are more stable and robust. Moreover, we believe that, by using orthogonal polynomials, strong noise in the PDF at low *r* can be reduced to a very low level without the need for hand-tuning of the number of polynomials to use. The problem with tuning by hand based on the user’s perception of the level of noise is that the end result depends on the user’s interpretation of an acceptable level of noise. We have seen many published PDFs where the user has tuned the number of polynomials to give a flat PDF at low *r* rather than the characteristic downwards slope. Anecdotally, we understand that many users are prepared to disregard the low-*r* part of the PDF, yet this is the region of the PDF where we can obtain an unambiguous determination of the normalization of the PDF, something that is essential if the areas of the peaks in the PDF are to be used to give quantitative information on coordination numbers.

We have demonstrated our approach using synthetic data, including testing the various points of implementation. We have shown that we can reliably reconstruct the PDF by fitting to the corresponding scattering function, including when the scattering function has been convolved with a resolution function. We have also explored the application of the ideas to real scattering data, both X-ray and neutron, for both amorphous and crystalline samples, taking account of resolution and extraneous contributions to the scattering function. The key issue with data from modern neutron facilities is that data from different banks of detectors have different resolution and often have inconsistent background levels. Our work shows that we can overcome these issues. Whereas in practice most scattering functions obtained from modern neutron facilities are stitched together without accounting for different resolution, and with *ad hoc* corrections made to account for the different background levels, our approach allows for a much more consistent way to merge data to obtain a best PDF without the user needing to intervene very much. We hope that we have demonstrated that this method works and that it is potentially useful in practice.

Nevertheless, the ambition of this paper has been limited to giving a proof-of-principle demonstration. It is necessary to confront a wider range of data, for both neutron and X-ray total scattering measurements. There may be some scope for improvement. For example, although it is not possible to simultaneously fit both the background Chebyshev polynomials and the Hermite functions, because the two types of function are not orthogonal with each other, it may be possible to have some constraints that can allow a degree of fitting of both with neutron scattering data, given that the same parts of the scattering function are found in different detectors.

We see one major use case being to prepare data for modelling such as with the RMC method (Tucker *et al.*, 2007 ▸). In this case, absolute accuracy is a necessity, and to date one major limitation is that it has not been possible to remove the effects of resolution from the scattering data and PDF. These can be accounted for approximately by modulating the calculated PDF by a Gaussian envelope function that is the Fourier transform of the resolution function (Sławiński *et al.*, 2024 ▸). However, this is clearly a gross – albeit relatively effective – approximation in that it ignores the *Q* dependence of the resolution function, and it ignores the fact that the PDF is formed from merging data sets of different resolution. By removing the effects of resolution from the PDF, there is no need to try to account for this within the RMC simulation. Furthermore, in RMC no account is taken of resolution in the use of the scattering function, but our method removes the resolution from the scattering function before it is used in the RMC method. Mostly we have focused attention here on the PDF, but being able to extract a scattering function free of the effects of resolution is equally important for application in the RMC method. We had at one point attempted to account for resolution exactly within the RMC process (Tucker *et al.*, 2007 ▸), but this was too expensive in terms of running time and therefore never used in practice.

For application in the RMC method, the approach of using equation (22) to account for the sharpness of the Bragg peaks actually causes no problem in the RMC analysis, because exactly the same approach is used there to account for the finite range of values of *r* caused by the size of the configuration (Nield *et al.*, 1992 ▸; McGreevy, 2001 ▸). That is, to account for the termination ripples caused by the Fourier transform of the PDF taken to distances limited by the size of the configuration, the derived scattering function contains termination ripples, which need to be ‘added’ to the scattering data. We are, in effect, preparing the scattering data for use in RMC if the same value of 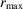 is to be used.

The method here has been implemented within the framework of MATLAB, and it would be quite easy to implement for any other programming language. In fact, we have also demonstrated that the method works with Fortran. With MATLAB the slowest procedure is the generation of the Hermite functions, and we have demonstrated a speed-up by converting this part of the script into the C language and using MATLAB’s tools to convert this into a compiled function.

## 9. Supporting information

Additional results for synthetic data are given in the supporting information. The main text reports synthetic data generated using the crystal of andalusite. Results corresponding to those shown in Figs. 2 ▸(*d*), 4 ▸ and 5 ▸ are presented also for crystals of calcite, acetylene, α-quartz and α-cristobalite. These simulations used the model interatomic potentials of Archer *et al.* (2003 ▸) and Peng *et al.* (2023 ▸), based on the parameters of Williams (2001 ▸) and Sanders *et al.* (1984 ▸).

## 10. Availability of data and software

The *HermitePDF* software and all test data are available from https://doi.org/10.5281/zenodo.14220369.

## Supplementary Material

This contains many figures that give additional examples of applications of the method and software. DOI: 10.1107/S1600576725004340/yr5150sup1.pdf

## Footnotes

1 The function defined here as  is given the symbol  by some communities. Keen (2001 ▸) has given a helpful comparison of different nomenclatures. The issue of naming the PDF has been discussed by Dove & Li (2022 ▸); our feeling is that the symbol  is better reserved for a combination of the partial functions  without the multiplication by *r*.

2 Note that Krylov & Vvedenskii (1995 ▸) did not use the scaling coefficient  in their application of this method; the limited range of *Q* in the data of their examples coupled with the limited range of *r* over which the PDF had some structure did not require this scaling to be carried out.

3 In the examples that we present in this paper we have tacitly avoided this problem, because here we formed synthetic scattering data for crystalline phases from the Fourier transform of synthetic PDFs that span only a finite range of distances. This in itself broadens the Bragg peaks, but we have broadened them further by modulating the PDF with the Lorch function as described below. These factors all led to us generating scattering data with broadened features.

4 To explain why, for a spallation neutron source, we expect that the resolution width , we note that there are two main sources of resolution broadening. One is the width in angular range of the detectors, which leads to a broadening that varies as , where θ and  differ for each bank of detectors. The other is uncertainty in the length of the neutron flight path *L* due to the finite thickness of the neutron moderator, with . Since in both cases the right-hand side is a constant, we have  to be constant to a good approximation.

## Appendix A Format of the input file for the *HermitePDF* program

The program has one main input file containing a set of commands to control its running. These are in the form of either a keyword directive (a simple statement) or keyword–value pairs. In each case, the keyword is required to be followed by two colons. The keywords can be given in any order.

### A1. Keyword directives

These keywords can have true or false values. However, the default if the line is not given is false, and the presence of the line without a value gives the default of true.

broaden_data :: Convolve the experimental data with an instrument resolution function. This is provided for testing with synthetic data and it is expected that most applications will not make use of this keyword.

convolution :: Convolve the data with a sinc function to better handle the sharp Bragg peaks [equation (22)].

fit_with_instrument_resolution :: Convolve the Hermite functions with an instrument resolution function and fit the experimental data with these broadened Hermite functions.

Gudrun_file :: Give the number of data banks in the *Gudrun* file, and for each *Gudrun* file give an additional line containing the name of the file.

Lorch :: Apply a Lorch correction to the experimental data to avoid termination ripples [equation (21)].

pre_fitting :: Pre-fit the experimental data with low-order odd-order Chebyshev polynomials, and subtract these from the data in order to level up the background levels.

print_graphs :: Print the resultant graphs to PDF files. Whilst this may seem an obvious thing to do, it does delay ending of the program by a noticeable amount.

### A2. Keyword/value pairs

broaden_information :: Give the name of the file containing the information for the resolution function parameters and resolution function type for experimental data in conjunction with the broaden_data keyword when used.

Chebyshev :: Give the number of Chebyshev polynomials to be used. Note that this is the total number used rather than the maximum order.

data_files :: Give the number of data files, and for each data file give, in additional lines, the number of the bank consistent with the number of the resolution parameter file (see the resolution_information keyword) and the names of the files containing the scattering data.

data_type :: Give the type of function for the experimental scattering data; options are Qi(Q) (default), i(Q) and S(Q).

nHermites :: Give the number of Hermite functions to be used [equation (20)]. Note that this is the total number used rather than the maximum order *n* given in equation (16). If the value of Rmax :: is also given, this line will be ignored, with the value being set automatically by equation (20).

PDF_file :: Give the name of the file containing the PDF to compare with the computed PDF.

Qfitmax :: Give the maximum value of *Q* to be used in the fitting.

Qfitmin :: Give the minimum value of *Q* to be used in the fitting.

resolution_information :: Give the name of the file containing the information for the resolution function parameters and resolution function type for Hermite functions.

Rmax :: Give the maximum value of *r* in the computed  function. This is not required if nHermites :: is also given, but if it is given it will make nHermites :: redundant because the number of Hermite functions will then be set by equation (20).

Rspacing :: Give the point spacing in the computed  function.

self_term :: Give the expected value of , which will be required for the conversion from  to . Give all values on the same line.

### A3. Example input file

Here is an example of an input file:[Chem scheme1]

### A4. Additional files 1. Data files

The default format for the data files is as follows:

Line 1: the number of data points.

Line 2: a title.

Subsequent lines: one pair of values per line, giving the value of *Q* and the corresponding value of the scattering function.

This file is specified by the Data_files :: keyword. The other type of data file is a *Gudrun* DSC output file, which contains 14 header lines each beginning with a # character, followed by a number of lines containing first the value of *Q* followed by pairs of  and  values for each bank in the raw data. In this case the Data_type :: keyword is not necessary. This is specified by the Gudrun_files :: keyword.

### A5. Additional files 2. Resolution function information files

This file type is required if either of the broaden_information or resolution_information key­words are given. In both cases the same format for the information files is used. The first line contains the type of the resolution function. This is specified by the resolution_type :: keyword. The subsequent lines contain the information for the resolution function parameters.

Resolution_type :: Give the type of the resolution function; options are Gaussian, pseudo_voigt, back_to_back and gaussian_tof.

Parameter :: This is in the form of keyword–value pairs. Give the type of the parameter before the colons. Options are:

(i) sigma for Resolution_type :: Gaussian.

(ii) sigma, gamma and ratio for Resolution_type :: pseudo_voigt.

(iii) sigma, gamma, ratio, lambda_up and lambda_down for Resolution_type :: back_to_back.

(iv) a and c for Resolution_type :: gaussian_tof (a and c are, respectively, the coefficients of the linear term and constant term of the *Q*-dependent sigma for a Gaussian).

If there are a set of data banks, add 1, 2 and 3 to the parameter to distinguish between different banks, like Parameter1 ::, Parameter2 :: and Parameter3 ::.

Note that each parameter takes up a separate row and the value of each parameter is placed behind the colons.

Here is an example of a file giving information for the resolution function:[Chem scheme2]

## Appendix B Notes on the implementation of *HermitePDF* in MATLAB

As noted earlier, this project has been implemented initially in MATLAB as our proof-of-concept design (see Section 10 for the availability of the *HermitePDF* software). There are two points in the implementation that are worth noting.

The first point is that, by using MATLAB’s backslash operator to find the set of  values that give best agreement with experimental data, we are working with linear equations. This means that we cannot in this implementation refine relative scale factors for different banks of detectors, for example. Another example might be to allow refinement of the resolution parameters. These will require an implementation using a different function minimization method, which is something that could be done in principle but was not attempted because the work presented here is primarily a proof of concept.

The second point is that the slowest part of our implementation is the calculation of the Hermite functions. This task is carried out in the function made available by Polkinghorne (2022 ▸), and the process is slow primarily because this function is called many times, with the time taken by each call arising from the fact that MATLAB needs to interpret the code line by line. MATLAB allows for C functions to be compiled, and so we have converted the function of Polkinghorne (2022 ▸) into C, and this gives a considerable gain in running speed.
