# Supplementary material for: Accounting for instrument resolution in the pair distribution functions obtained from total scattering data using Hermite functions
Source: J Appl Crystallogr. 2025 Jul 22;58(Pt 4):1269–87. doi: 10.1107/S1600576725004340 (PMC12321014; doi:10.1107/S1600576725004340)
Supplement: Supplementary file 1 [file j-58-01269-sup1.pdf]

JOURNAL OF APPLIED CRYSTALLOGRAPHY

Volume 58 (2025)

Supporting information for article:

Accounting for instrument resolution in the pair distribution functions obtained from total scattering data using Hermite functions

Shaojie Wang, Min Gao, Yinze Qin, Sijie Zhang, Lei Tan and Martin Dove

Accounting for instrument resolution in the pair distribution functions obtained from total scattering data using Hermite functions

Shaojie Wang [a], Min Gao [b,c], Yinze Qin [d], Sijie Zhang [d,e], Lei Tan [f], Martin T Dove [a,e,g,*]

[a] *Institute of Atomic and Molecular Physics, Sichuan University, Chengdu, Sichuan, 610065, China*

[b] *CrystalMaker Software Ltd, Centre for Innovation & Enterprise, Oxford University Begbroke Science Park, Woodstock Road, Begbroke, Oxfordshire, OX5 1PF, United Kingdom*

[c] *Some work performed at: China Spallation Neutron Source, Institute of High Energy Physics, Chinese Academy of Sciences, 1 Zhongziyuan Road, Dongguan, 523803, China*

[d] *College of Physics, Sichuan University, Chengdu, Sichuan, 610065, China*

[e] *School of Mechanical Engineering, Guizhou University of Engineering Science, Xueyan Road, Bijie, Guizhou, 55170, China*

[f] *Department of Physics, School of Physics and Mechanics, Wuhan University of Technology, Wuhan, Hubei, 430070, China*

[g] *School of Physical and Chemical Sciences, Queen Mary University of London, Mile End Road, London, E1 4NS, United Kingdom*

* *Corresponding author, email: martin.dove@icloud.com*

Abstract

This document contains supplementary data for the paper.

S1. Testing with synthetic data 1: basic fitting to scattering function

Here we present several examples of fitting a synthetic $Qi(Q)$ function by Hermite functions, and forming the $D(r)$ function by recombining the Hermite functions to produce the Fourier transform. These examples support the discussion in Section 4.3 of the main paper.

The initial PDFs have been formed using a special module of the lattice simulation program GULP (Gale (1997); Gale and Rohl (2003); Cope and Dove (2007)).

For each example we show the a) synthetic scattering function fitted to Hermite functions, and b) the corresponding $D(r)$ obtained from this, compared with the original PDF formed from lattice simulations. The only differences between the extracted form of $D(r)$ and that obtained form the initial lattice simulation arises from the use of the modification function in forming the initial Fourier transform of the PDF to compute the synthetic scattering function. In each case $Q_{\max} = 50$ Å^{-1} and $r_{\max} = 50$ Å. In each case we used the neutron scattering lengths.

The data we show here are

Figure S1: Andalusite, Al_2SiO_5 (orthorhombic space group $Pnnm$), simulated using the interatomic potential energy model described by Winkler et al. (1991), based on an initial model for silica by Sanders et al. (1984).

Figure S2: Calcite, $CaCO_3$ (rhombohedral space group $R\overline{3}m$), simulated using the interatomic potential energy model of Archer et al. (2003).

Figure S3: Acetylene, C_2H_4 (orthorhombic space group $Cmce$), simulated using the interatomic potential energy model of Williams (2001), with atomic charges as used by Peng et al. (2023). In this case notice the negative peaks associated with the negative scattering length of hydrogen (corresponding to C–H distances).

Figure S4: α-Quartz, SiO_2 (trigonal space group $P3_221$), simulated using the interatomic potential energy model of Sanders et al. (1984).

Figure S5: α-Cristobalite, SiO_2 (tetragonal space group, $P4_12_12$), simulated using the interatomic potential energy model of Sanders et al. (1984).

In each example, the figure labelled (a) shows the simulated scattering function $Qi(Q)$ (black) fitted by the Hermite functions (red), and the figure labelled (b) gives a comparison of the initial simulated PDF (black) with the PDF reconstructed from the Hermite functions (red). In many cases the data (black) are almost completely overlapped by the curves obtained from the Hermite functions (red).

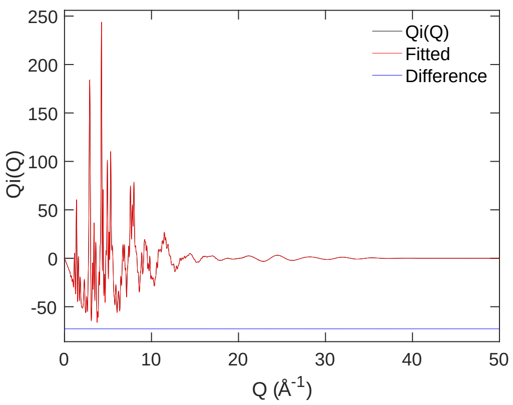

(a) Scattering function

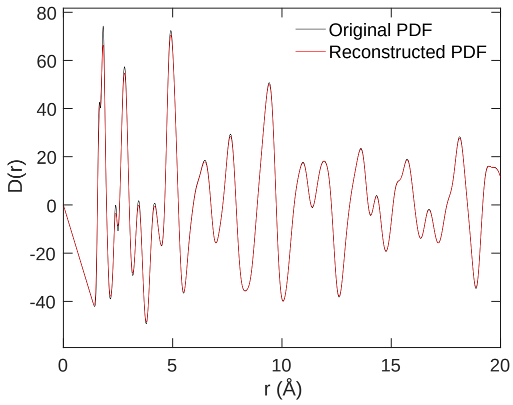

(b) PDF

Figure S1: (a) The fitted $Qi(Q)$ function obtained from the simulations of **andalusite**, Al_2SiO_5, with red showing the fitted curve (equation 16 in the main paper) and black showing the simulated data. (b) The resultant PDF $D(r)$ obtained by recombining the fitted Hermite functions (equation 17 in the main paper) shown as red, with black showing the original PDF from the lattice simulation.

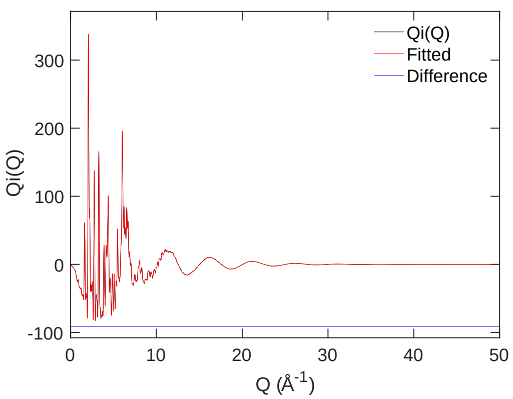

(a) Scattering function

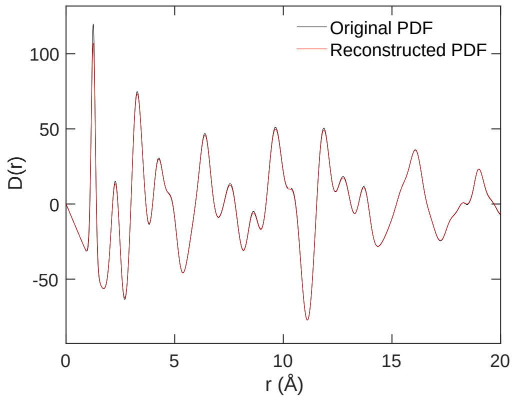

(b) PDF

Figure S2: (a) The fitted $Qi(Q)$ function obtained from the simulations of **calcite**, $CaCO_3$, with red showing the fitted curve (equation 16 in the main paper) and black showing the simulated data. (b) The resultant PDF $D(r)$ obtained by recombining the fitted Hermite functions (equation 17 in the main paper) shown as red, with black showing the original PDF from the lattice simulation.

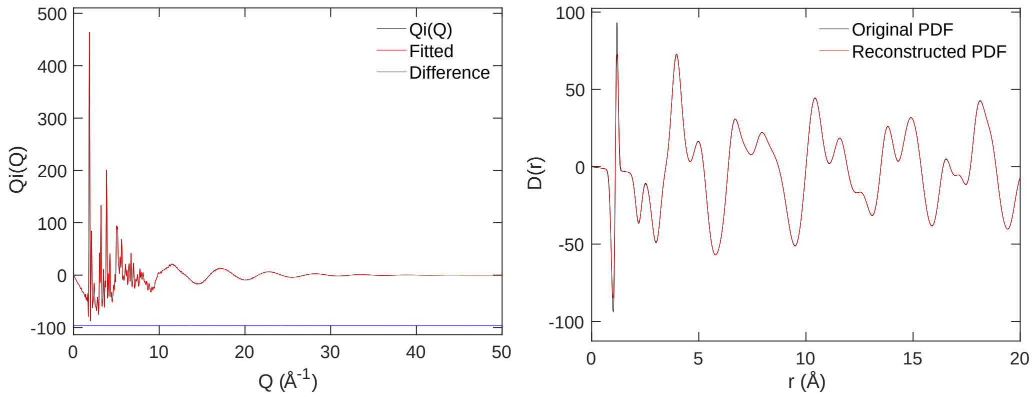

(a) Scattering function (b) PDF

Figure S3: (a) The fitted $Qi(Q)$ function obtained from the simulations of **acetylene**, C_2H_2, with red showing the fitted curve (equation 16 in the main paper) and black showing the simulated data. (b) The resultant PDF $D(r)$ obtained by recombining the fitted Hermite functions (equation 17 in the main paper) shown as red, with black showing the original PDF from the lattice simulation.

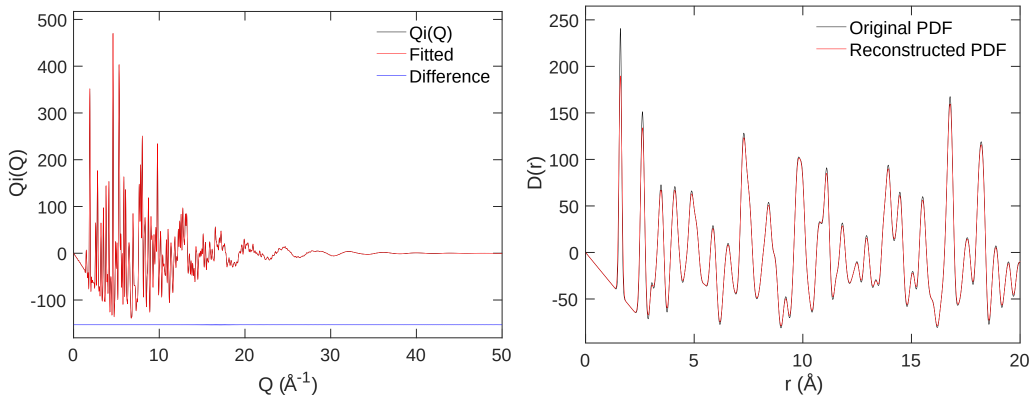

(a) Scattering function (b) PDF

Figure S4: (a) The fitted $Qi(Q)$ function obtained from the simulations of **α-quartz**, SiO_2, with red showing the fitted curve (equation 16 in the main paper) and black showing the simulated data. (b) The resultant PDF $D(r)$ obtained by recombining the fitted Hermite functions (equation 17 in the main paper) shown as red, with black showing the original PDF from the lattice simulation.

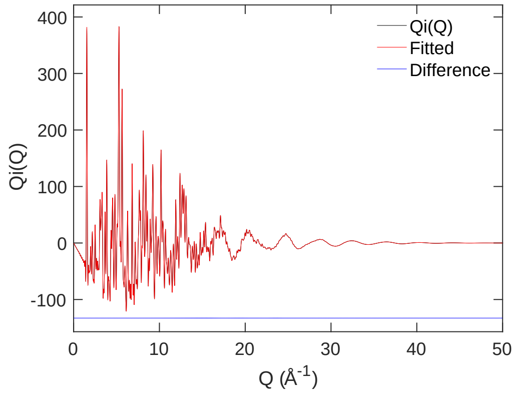

(a) Scattering function

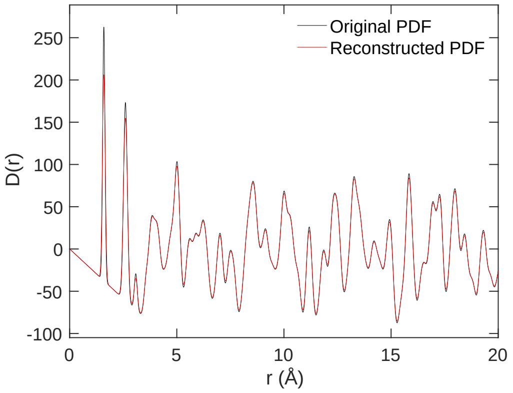

(b) PDF

Figure S5: (a) The fitted $Qi(Q)$ function obtained from the simulations of **α-cristobalite,** SiO_2, with red showing the fitted curve (equation 16 in the main paper) and black showing the simulated data. (b) The resultant PDF $D(r)$ obtained by recombining the fitted Hermite functions (equation 17 in the main paper) shown as red, with black showing the original PDF from the lattice simulation.

S2. Testing with synthetic data 2: Examples of fitting to scattering function with simulated instrument resolution

Here we present several examples of working with synthetic data for the scattering function $Qi(Q)$ that have been broadened by a resolution function, equation 24 in the main paper, as might be encountered in experimental measurements. We have fitted with Hermite functions that have been broadened by the same resolution function, equation 25 in the main paper, as shown in the graphs labelled (a) in each case, where the black curves represent the simulated data and the red curves represent the fitted function. We then obtained the underlying scattering function by using the pure Hermite functions (no broadening), as in equation 16 in the main paper, shown in the graphs labelled (b) in each case, where the black curves represent the simulated scattering function prior to broadening and the red curves represent the reconstructed scattering function. In this case each result is nearly identical, as was hoped. Finally we have formed the $D(r)$ functions by recombining the Hermite functions, equation 17 in the main paper, shown in the graphs labelled (c) in each case, where the black curves represent the simulated PDF and the red curves show the reconstructed functions.

We have considered the Gaussian resolution function discussed in the main paper (equation 26), together with two other resolution functions we describe here:

Pseudo-Voigt function: This is written as a linear combination of Gaussian and Lorenztian functions,

$$V(Q-Q_0) = \eta G(Q-Q_0) + (1-\eta)L(Q-Q_0) \tag{S1}$$

with the Lorentzian contribution defined as

$$L(Q-Q_0) = \frac{1}{\pi}\frac{\gamma}{(Q-Q_0)^2+\gamma^2} \tag{S2}$$

where $G(Q-Q_0)$ has been defined in the main paper (equation 26).

Back-to-back exponential function: One model of the lineshape in time-of-flight neutrons from spallation sources is to convolve two exponential functions, placed back-to-back, with a pseudo-Voigt function. This can be defined in more detail than considered here, but the general idea is common to such line shapes. The function is described as

$$R(Q-Q_0) = [\eta G(Q-Q_0) + (1-\eta)L(Q-Q_0)] \otimes E(Q-Q_0) \tag{S3}$$

where the pseudo-Voigt function was defined above, and we have the back-to-back exponential function

$$E(Q-Q_0)=\begin{cases}2N\exp\left(\alpha(Q-Q_0)\right), & \text{for } Q\leq Q_0\\ 2N\exp\left(-\beta(Q-Q_0)\right), & \text{for } Q>Q_0\end{cases} \tag{S4}$$

with

$$N=\frac{\alpha\beta}{2(\alpha+\beta)} \tag{S5}$$

In each case here, Q_{max} = 50 Å^{-1} and r_{max} = 50 Å. All parameters in the following equations, with the exception of η (dimensionless), have units of Å^{-1}.

The data we show here are

Figure S6: Andalusite (see Figure S1) with a pseudo-Voigt resolution function.

Figure S7: Andalusite (see Figure S1) with a back-to-back exponential resolution function.

Figure S8: Calcite (see Figure S2) with a Gaussian resolution function.

Figure S9: Calcite (see Figure S2) with a pseudo-Voigt resolution function.

Figure S10: Calcite (see Figure S2) with a back-to-back exponential resolution function.

Figure S11: Acetylene (see Figure S3) with a Gaussian resolution function.

Figure S12: Acetylene (see Figure S3) with a pseudo-Voigt resolution function.

Figure S13: Acetylene (see Figure S3) with a back-to-back exponential resolution function.

Figure S14: α-Quartz (see Figure S4) with a Gaussian resolution function.

Figure S15: α-Quartz (see Figure S4) with a pseudo-Voigt resolution function.

Figure S16: α-Quartz (see Figure S4) with a back-to-back exponential resolution function.

Figure S17: α-Cristobalite (see Figure S5) with a Gaussian resolution function.

Figure S18: α-Cristobalite (see Figure S5) with a pseudo-Voigt resolution function.

Figure S19: α-Cristobalite (see Figure S5) with a back-to-back exponential resolution function.

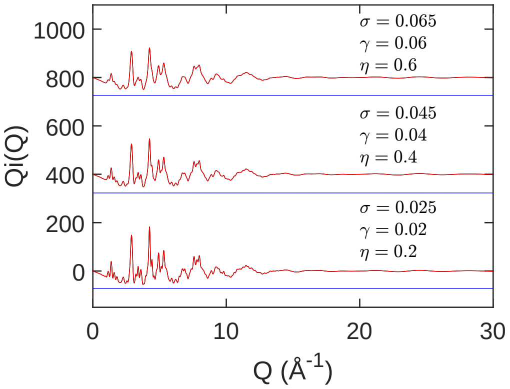

(a) Broadened scattering function

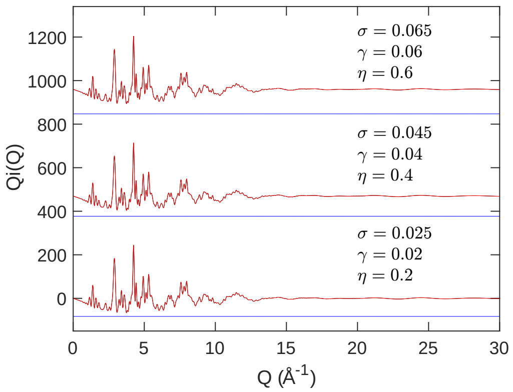

(b) Reconstructed scattering function

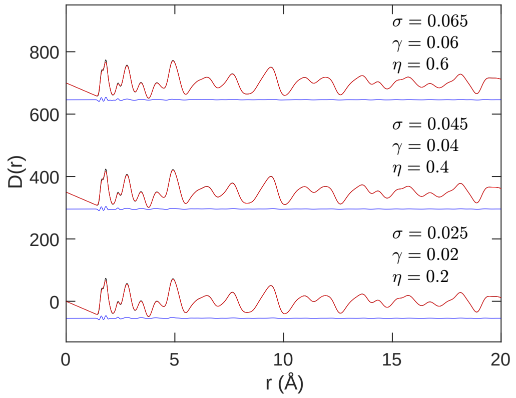

(c) PDF

Figure S6: a) The fitted $Qi(Q)$ function for the model **andalusite**, Al_2SiO_5, broadened by a pseudo-Voigt resolution function; b) the reconstructed $Qi(Q)$ function for the same model without broadening, with red showing the scattering function and black (which is almost completely overlapped by the red curve) showing the original simulated data; c) The reconstructed PDF $D(r)$ obtained from transform of the fitted $Qi(Q)$ function (equation 17) shown as red, with black showing the original PDF from the simulation configuration.

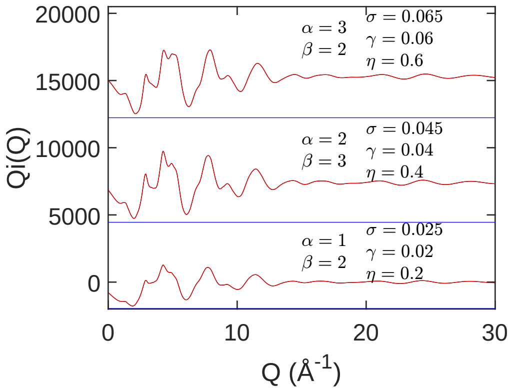

(a) Broadened scattering function

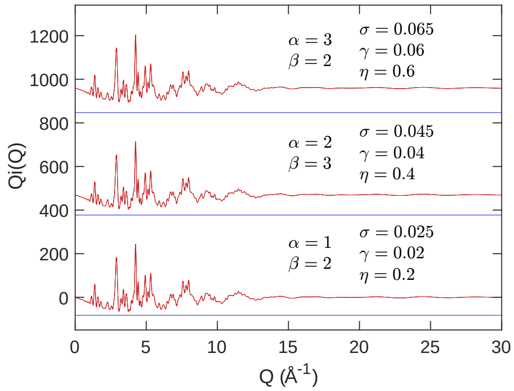

(b) Reconstructed scattering function

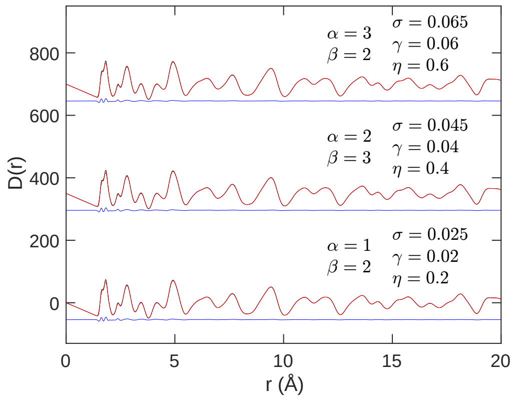

(c) PDF

Figure S7: a) The fitted $Qi(Q)$ function for the model **andalusite**, Al_2SiO_5, broadened by a resolution function that is constructed by convolving a back-to-back exponential function with a pseudo-Voigt function; b) the reconstructed $Qi(Q)$ function for the same model without broadening, with red showing the scattering function and black (which is almost completely overlapped by the red curve) showing the original simulated data; c) The reconstructed PDF $D(r)$ obtained from transform of the fitted $Qi(Q)$ function (equation 17) shown as red, with black showing the original PDF from the simulation configuration.

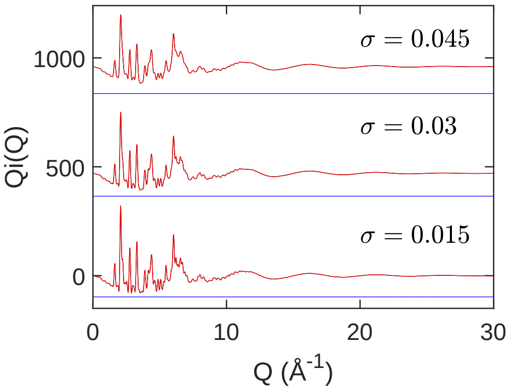

(a) Broadened scattering function

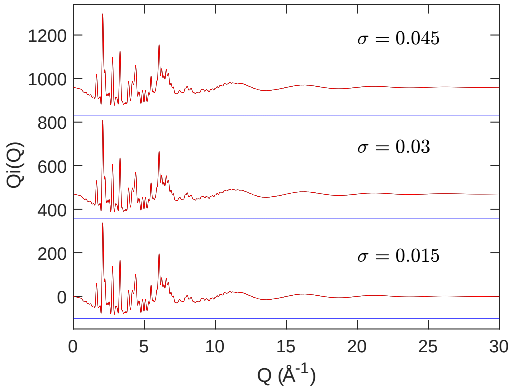

(b) Reconstructed scattering function

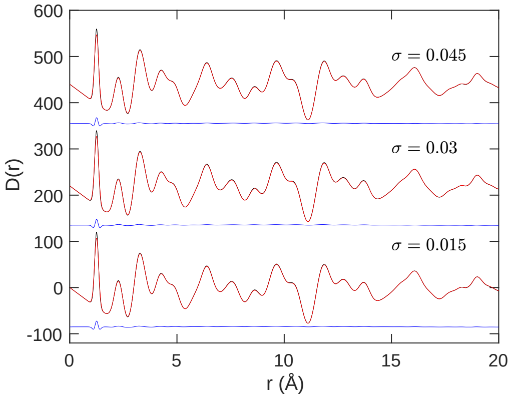

(c) PDF

Figure S8: a) The fitted $Qi(Q)$ function for the model **calcite**, $CaCO_3$, broadened by a Gaussian resolution function; b) the reconstructed $Qi(Q)$ function for the same model without broadening, with red showing the scattering function and black (which is almost completely overlapped by the red curve) showing the original simulated data; c) The reconstructed PDF $D(r)$ obtained from transform of the fitted $Qi(Q)$ function (equation 17) shown as red, with black showing the original PDF from the simulation configuration.

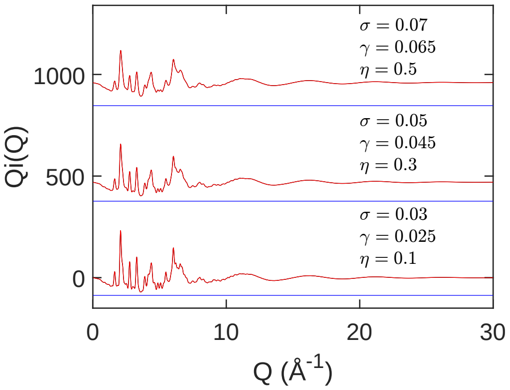

(a) Broadened scattering function

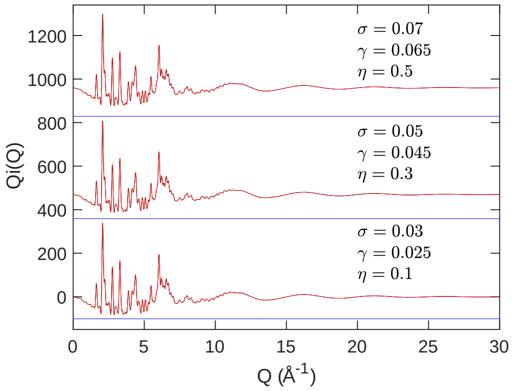

(b) Reconstructed scattering function

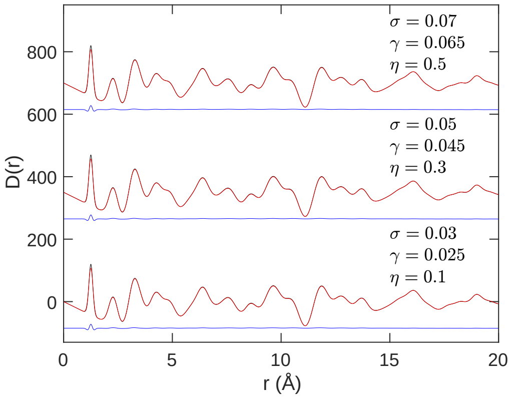

(c) PDF

Figure S9: a) The fitted $Qi(Q)$ function for the model **calcite**, $CaCO_3$, broadened by a pseudo-Voigt resolution function; b) the reconstructed $Qi(Q)$ function for the same model without broadening, with red showing the scattering function and black (which is almost completely overlapped by the red curve) showing the original simulated data; c) The reconstructed PDF $D(r)$ obtained from transform of the fitted $Qi(Q)$ function (equation 17) shown as red, with black showing the original PDF from the simulation configuration.

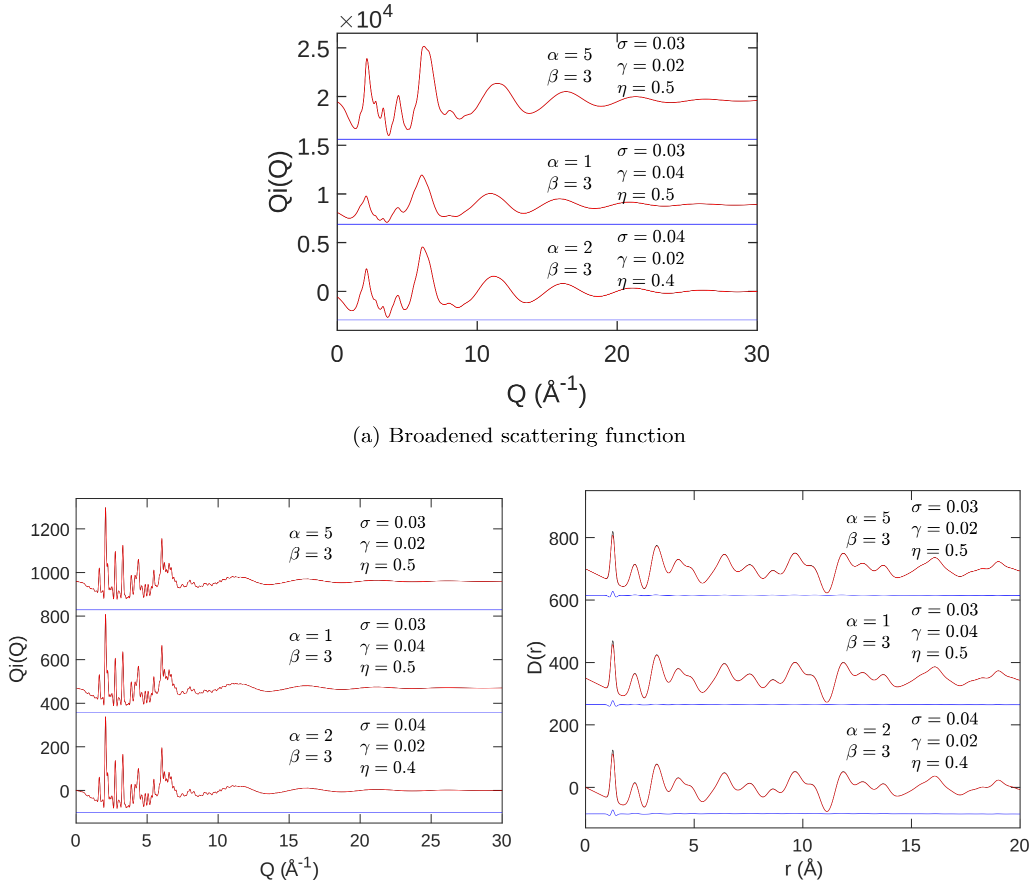

(a) Broadened scattering function

(b) Reconstructed scattering function

(c) PDF

Figure S10: a) The fitted $Qi(Q)$ function for the model **calcite**, $CaCO_3$, broadened by a resolution function that is constructed by convolving a back-to-back exponential function with a pseudo-Voigt function; b) the reconstructed $Qi(Q)$ function for the same model without broadening, with red showing the scattering function and black (which is almost completely overlapped by the red curve) showing the original simulated data; c) The reconstructed PDF $D(r)$ obtained from transform of the fitted $Qi(Q)$ function (equation 17) shown as red, with black showing the original PDF from the simulation configuration.

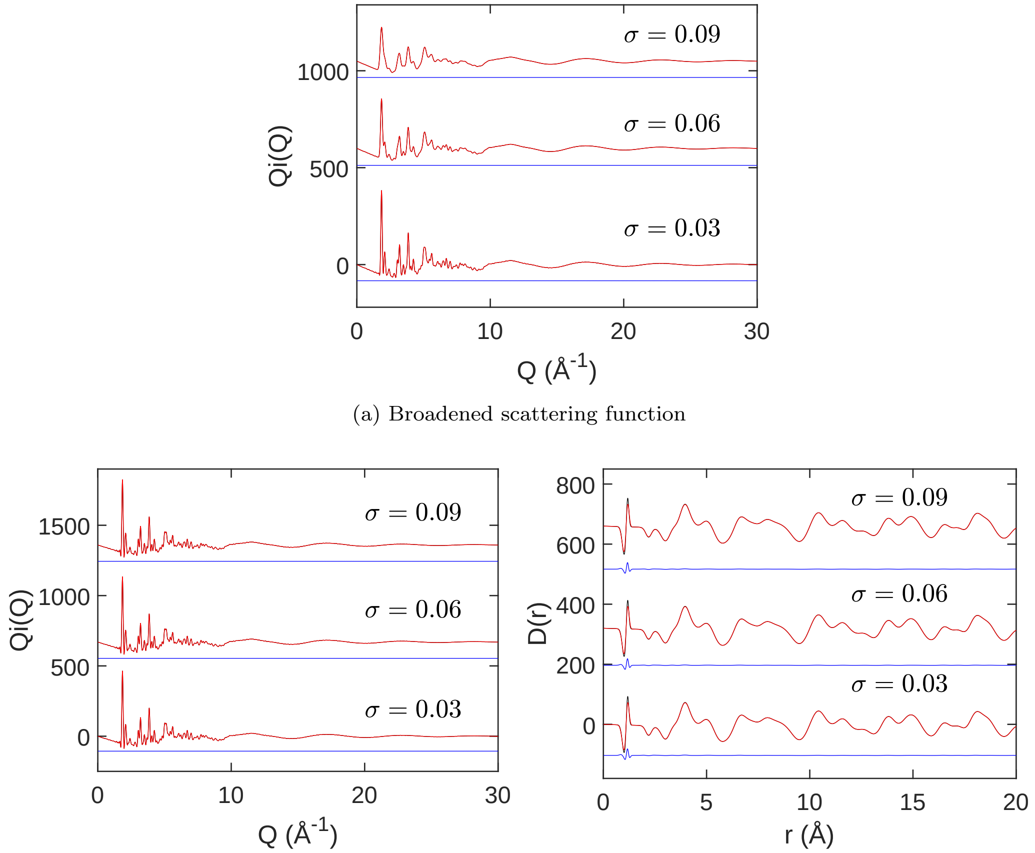

(a) Broadened scattering function

(b) Reconstructed scattering function

(c) PDF

Figure S11: a) The fitted $Qi(Q)$ function for the model **acetylene**, C_2H_2, broadened by a Gaussian resolution function; b) the reconstructed $Qi(Q)$ function for the same model without broadening, with red showing the scattering function and black (which is almost completely overlapped by the red curve) showing the original simulated data; c) The reconstructed PDF $D(r)$ obtained from transform of the fitted $Qi(Q)$ function (equation 17) shown as red, with black showing the original PDF from the simulation configuration.

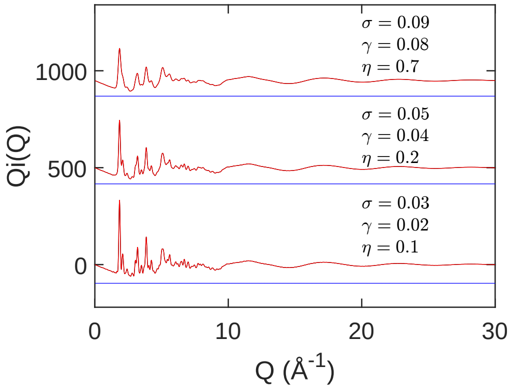

(a) Broadened scattering function

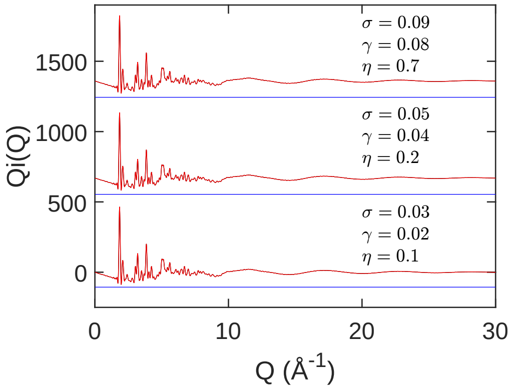

(b) Reconstructed scattering function

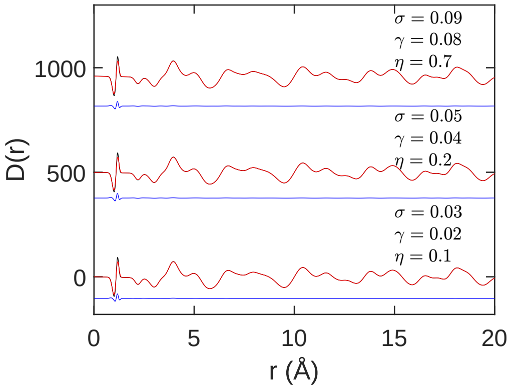

(c) PDF

Figure S12: a) The fitted $Qi(Q)$ function for the model **acetylene**, C_2H_2, broadened by a pseudo-Voigt resolution function; b) the reconstructed $Qi(Q)$ function for the same model without broadening, with red showing the scattering function and black (which is almost completely overlapped by the red curve) showing the original simulated data; c) The reconstructed PDF $D(r)$ obtained from transform of the fitted $Qi(Q)$ function (equation 17) shown as red, with black showing the original PDF from the simulation configuration.

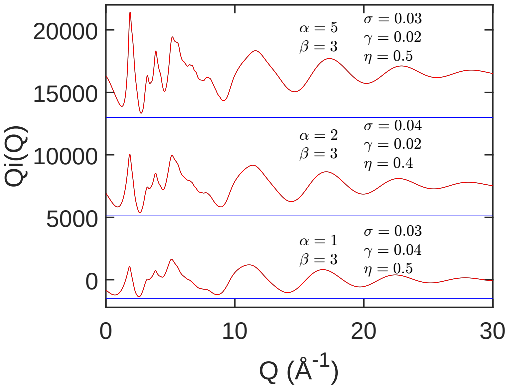

(a) Broadened scattering function

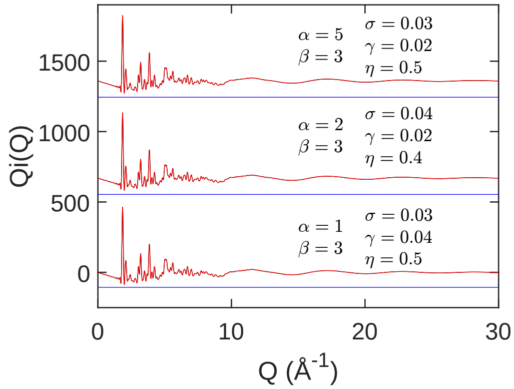

(b) Reconstructed scattering function

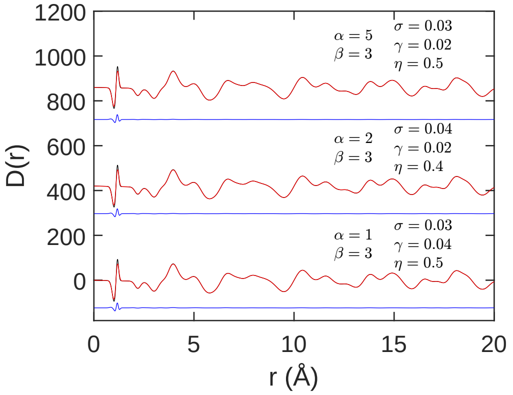

(c) PDF

Figure S13: a) The fitted $Qi(Q)$ function for the model **acetylene**, C_2H_2, broadened by a resolution function that is constructed by convolving a back-to-back exponential function with a pseudo-Voigt function; b) the reconstructed $Qi(Q)$ function for the same model without broadening, with red showing the scattering function and black (which is almost completely overlapped by the red curve) showing the original simulated data; c) The reconstructed PDF $D(r)$ obtained from transform of the fitted $Qi(Q)$ function (equation 17) shown as red, with black showing the original PDF from the simulation configuration.

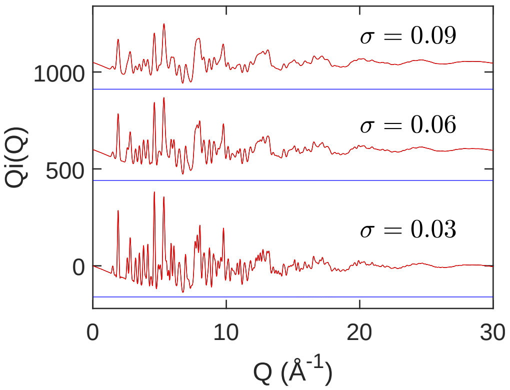

(a) Broadened scattering function

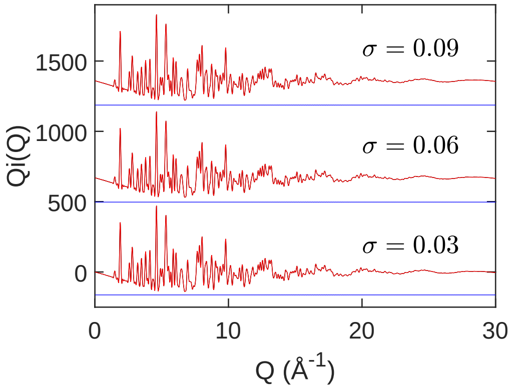

(b) Reconstructed scattering function

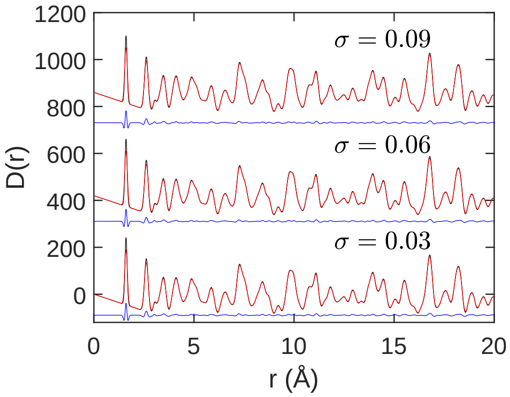

(c) PDF

Figure S14: a) The fitted $Qi(Q)$ function for the model **α-quartz**, SiO_2, broadened by a Gaussian resolution function; b) the reconstructed $Qi(Q)$ function for the same model without broadening, with red showing the scattering function and black (which is almost completely overlapped by the red curve) showing the original simulated data; c) The reconstructed PDF $D(r)$ obtained from transform of the fitted $Qi(Q)$ function (equation 17) shown as red, with black showing the original PDF from the simulation configuration.

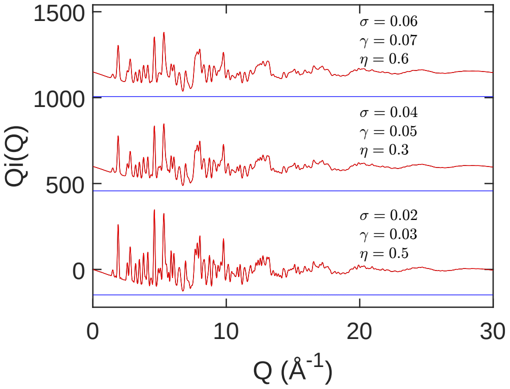

(a) Broadened scattering function

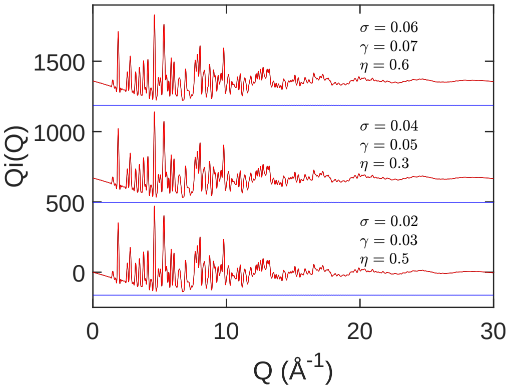

(b) Reconstructed scattering function

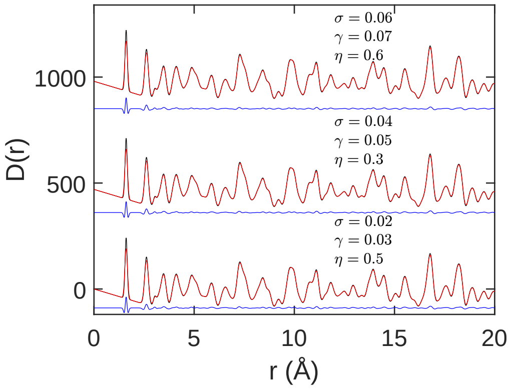

(c) PDF

Figure S15: a) The fitted $Qi(Q)$ function for the model **α-quartz**, SiO_2, broadened by a pseudo-Voigt resolution function; b) the reconstructed $Qi(Q)$ function for the same model without broadening, with red showing the scattering function and black (which is almost completely overlapped by the red curve) showing the original simulated data; c) The reconstructed PDF $D(r)$ obtained from transform of the fitted $Qi(Q)$ function (equation 17) shown as red, with black showing the original PDF from the simulation configuration.

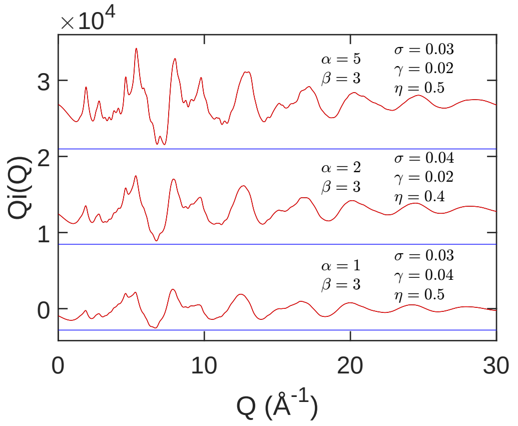

(a) Broadened scattering function

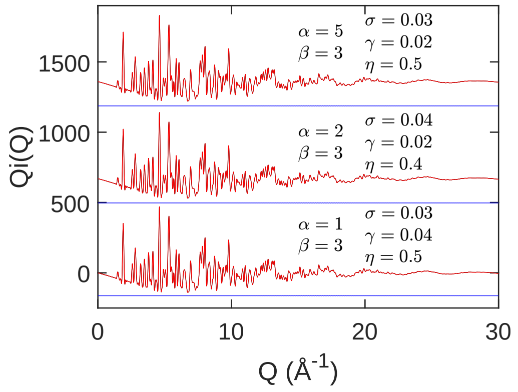

(b) Reconstructed scattering function

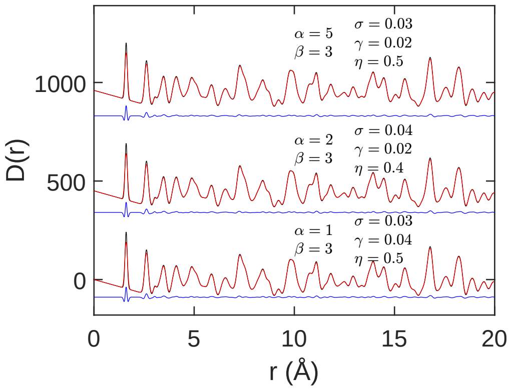

(c) PDF

Figure S16: a) The fitted $Qi(Q)$ function for the model **α-quartz**, SiO_2, broadened by a resolution function that is constructed by convolving a back-to-back exponential function with a pseudo-Voigt function; b) the reconstructed $Qi(Q)$ function for the same model without broadening, with red showing the scattering function and black (which is almost completely overlapped by the red curve) showing the original simulated data; c) The reconstructed PDF $D(r)$ obtained from transform of the fitted $Qi(Q)$ function (equation 17) shown as red, with black showing the original PDF from the simulation configuration.

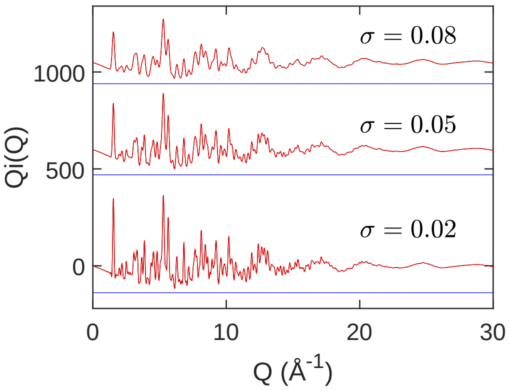

(a) Broadened scattering function

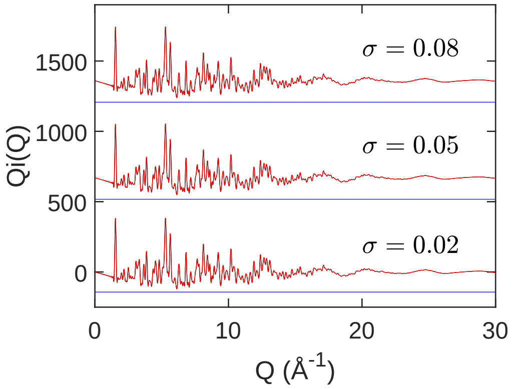

(b) Reconstructed scattering function

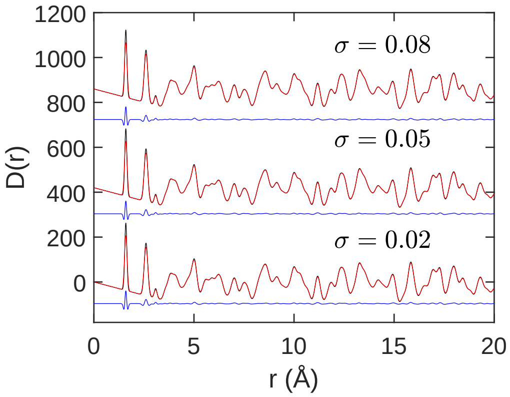

(c) PDF

Figure S17: a) The fitted $Qi(Q)$ function for the model ***α*-cristobalite**, SiO_2, broadened by a Gaussian resolution function; b) the reconstructed $Qi(Q)$ function for the same model without broadening, with red showing the scattering function and black (which is almost completely overlapped by the red curve) showing the original simulated data; c) The reconstructed PDF $D(r)$ obtained from transform of the fitted $Qi(Q)$ function (equation 17) shown as red, with black showing the original PDF from the simulation configuration.

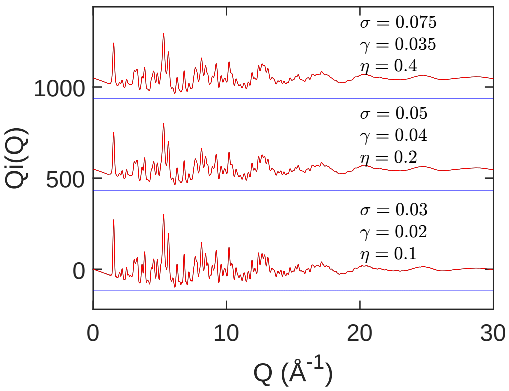

(a) Broadened scattering function

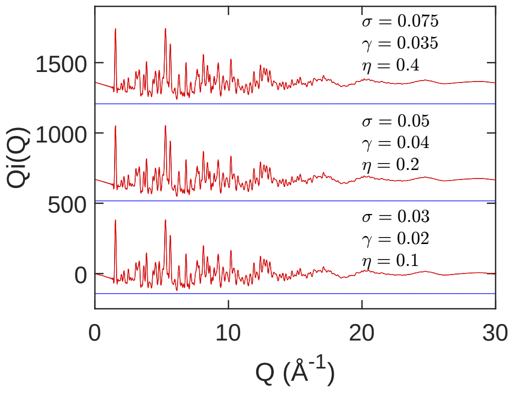

(b) Reconstructed scattering function

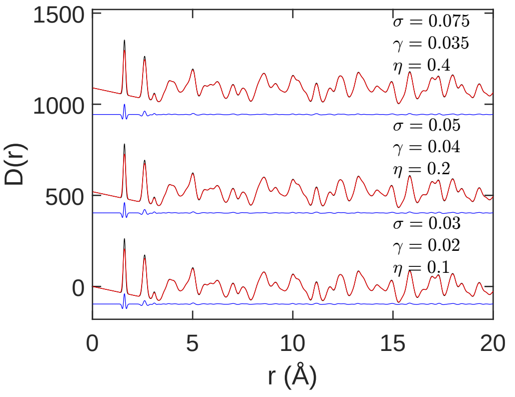

(c) PDF

Figure S18: a) The fitted $Qi(Q)$ function for the model **α-cristobalite**, SiO_2, broadened by a pseudo-Voigt resolution function; b) the reconstructed $Qi(Q)$ function for the same model without broadening, with red showing the scattering function and black (which is almost completely overlapped by the red curve) showing the original simulated data; c) The reconstructed PDF $D(r)$ obtained from transform of the fitted $Qi(Q)$ function (equation 17) shown as red, with black showing the original PDF from the simulation configuration.

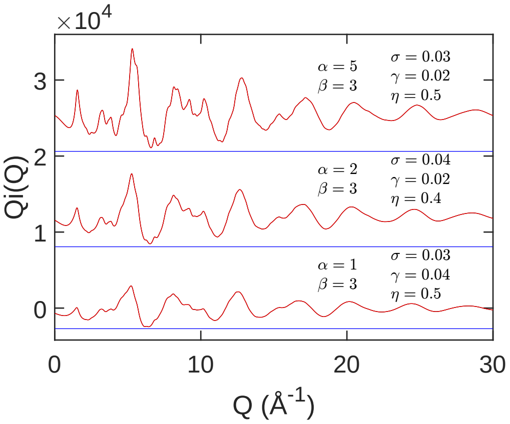

(a) Broadened scattering function

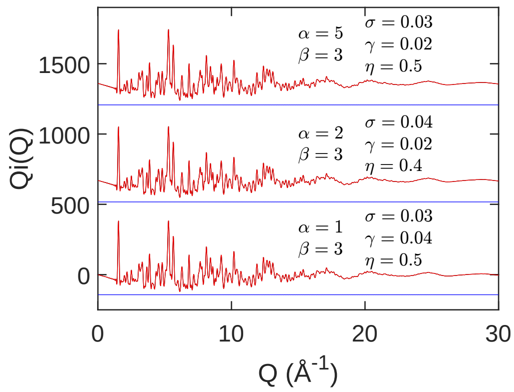

(b) Reconstructed scattering function

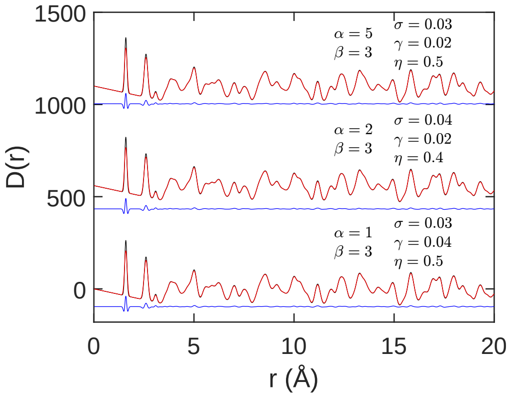

(c) PDF

Figure S19: a) The fitted $Qi(Q)$ function for the model **α-cristobalite**, SiO_2, broadened by a resolution function that is constructed by convolving a back-to-back exponential function with a pseudo-Voigt function; b) the reconstructed $Qi(Q)$ function for the same model without broadening, with red showing the scattering function and black (which is almost completely overlapped by the red curve) showing the original simulated data; c) The reconstructed PDF $D(r)$ obtained from transform of the fitted $Qi(Q)$ function (equation 17) shown as red, with black showing the original PDF from the simulation configuration.

S3. Testing with synthetic data 3: Demonstration of the effects of simulated instrument resolution with wrong parameters

In this section, we demonstrate the effects of using different parameter values for resolution function, resulting in three scenarios:

1. over-compensation of the effects of resolution, leading to a PDF with larger amplitude at higher values of r;
2. use of the appropriate parameters in the resolution function, giving good agreement between the reconstructed and original PDF;
3. taking no account of the resolution, leading to a PDF with smaller amplitude at higher values of r.

Figure 5 of the main paper shows results for andalusite with a Gaussian resolution function. Here we show

Figure S20: Simulation for andalusite (Figure S1) with pseudo-Voigt resolution function.

Figure S21: Simulation for calcite (Figure S2) with Gaussian resolution function.

Figure S22: Simulation for calcite (Figure S2) with pseudo-Voigt resolution function.

In each graph the values of the parameters of the resolution function are given.

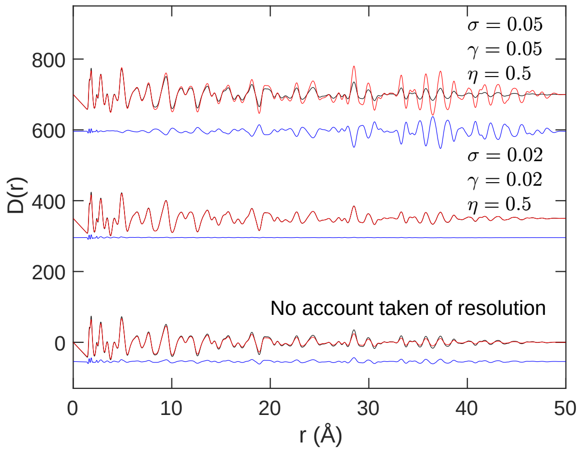

Figure S20: Demonstration of the effects of using the wrong parameter when fitting using a pseudo-Voigt resolution function, simulated using synthetic data for **andalusite**. In each case the black curve represents the original synthetic $D(r)$ function, the red curve represents the reconstructed PDF, and the blue curve represents the difference.

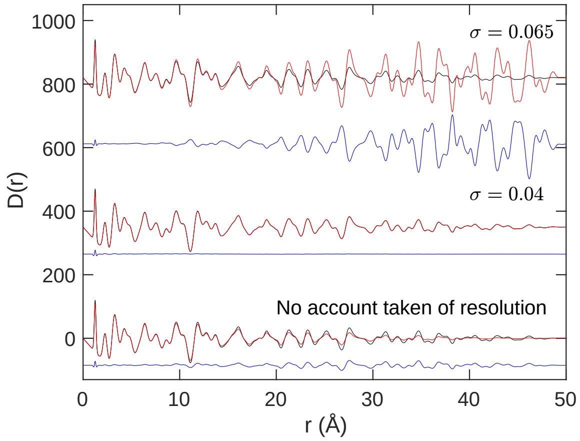

Figure S21: Demonstration of the effects of using the wrong parameter when fitting using a Gaussian resolution function, simulated using synthetic data for **calcite**. In each case the black curve represents the original synthetic $D(r)$ function, the red curve represents the reconstructed PDF, and the blue curve represents the difference.

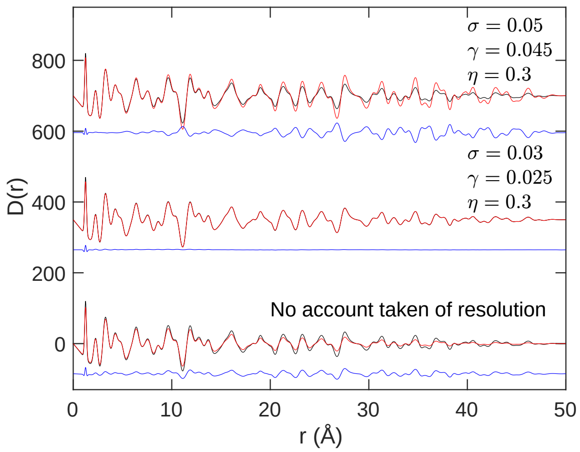

Figure S22: Demonstration of the effects of using the wrong parameter when fitting using a pseudo-Voigt resolution function, simulated using synthetic data for **calcite**. In each case the black curve represents the original synthetic $D(r)$ function, the red curve represents the reconstructed PDF, and the blue curve represents the difference.

References

Archer, T.D., Birse, S.E.A., Dove, M.T., Redfern, S.A.T., Gale, J.D., Cygan, R., 2003. An interatomic potential model for carbonates allowing for polarization effects. Physics and Chemistry of Minerals 30, 416–424. doi:10.1007/s00269-002-0269-z.

Cope, E.R., Dove, M.T., 2007. Pair distribution functions calculated from interatomic potential models using the *General Utility Lattice Program*. Journal of Applied Crystallography 40, 589–594. doi:10.1107/S0021889807016032.

Gale, J.D., 1997. GULP: A computer program for the symmetry-adapted simulation of solids. Journal of the Chemical Society, Faraday Transactions 93, 629–637. doi:10.1039/a606455h.

Gale, J.D., Rohl, A.L., 2003. The General Utility Lattice Program (GULP). Molecular Simulation 29, 291–341. doi:10.1080/0892702031000104887.

Peng, J., Zhang, S., Refson, K., Dove, M.T., 2023. Unique features of the structural phase transition in acetylene showing simultaneous characteristics of reconstructive, displacive and order–disorder. Physical Chemistry Chemical Physics 25, 9909–9924. doi:10.1039/d3cp00400g.

Sanders, M.J., Leslie, M., Catlow, C.R.A., 1984. Interatomic potentials for SiO_2. Journal of the Chemical Society, Chemical Communications , 1271–1273doi:10.1039/c39840001271.

Williams, D.E., 2001. Improved intermolecular force field for molecules containing H, C, N, and O atoms, with application to nucleoside and peptide crystals. Journal of Computational Chemistry 22, 1154–1166. doi:10.1002/jcc.1074.

Winkler, B., Dove, M.T., Leslie, M., 1991. Static lattice energy minimization and lattice dynamics calculations on aluminosilicate minerals. American Mineralogist 76, 313–331.
